# Cognitive tasks and combined statistical methods to evaluate, model, and predict mental workload

**DOI:** 10.3389/fpsyg.2023.1122793

**Published:** 2023-05-12

**Authors:** Lina-Estelle Linelle Louis, Saïd Moussaoui, Aurélien Van Langhenhove, Sébastien Ravoux, Thomas Le Jan, Vincent Roualdes, Isabelle Milleville-Pennel

**Affiliations:** ^1^Entreprise Onepoint, Nantes, France; ^2^École Centrale Nantes, CNRS, LS2N, UMR 6004, Nantes Université, Nantes, France; ^3^Department of Neurosurgery, CHU (Centre Hospitalier et Universitaire) Nord Laënnec, Saint-Herblain, France

**Keywords:** mental workload (MWL), NASA-TLX, workload profile, cognitive tasks, performances, K-means (KM) clustering, Linear Discriminant Analysis (LDA), permutation feature importance

## Abstract

Mental workload (MWL) is a concept that is used as a reference for assessing the mental cost of activities. In recent times, challenges related to user experience are determining the expected MWL value for a given activity and real-time adaptation of task complexity level to achieve or maintain desired MWL. As a consequence, it is important to have at least one task that can reliably predict the MWL level associated with a given complexity level. In this study, we used several cognitive tasks to meet this need, including the N-Back task, the commonly used reference test in the MWL literature, and the Corsi test. Tasks were adapted to generate different MWL classes measured *via* NASA-TLX and Workload Profile questionnaires. Our first objective was to identify which tasks had the most distinct MWL classes based on combined statistical methods. Our results indicated that the Corsi test satisfied our first objective, obtaining three distinct MWL classes associated with three complexity levels offering therefore a reliable model (about 80% accuracy) to predicted MWL classes. Our second objective was to achieve or maintain the desired MWL, which entailed the use of an algorithm to adapt the MWL class based on an accurate prediction model. This model needed to be based on an objective and real-time indicator of MWL. For this purpose, we identified different performance criteria for each task. The classification models obtained indicated that only the Corsi test would be a good candidate for this aim (more than 50% accuracy compared to a chance level of 33%) but performances were not sufficient to consider identifying and adapting the MWL class online with sufficient accuracy during a task. Thus, performance indicators require to be complemented by other types of measures like physiological ones. Our study also highlights the limitations of the N-back task in favor of the Corsi test which turned out to be the best candidate to model and predict the MWL among several cognitive tasks.

## Introduction

### Conceptual framework of mental workload

Mental workload (MWL) was first introduced by Bornemann (1942) aiming at optimizing human-machine systems. Though several definitions of MWL have been proposed since then, they all concur that MWL is a *multidimensional concept* (Hancock et al., 2021). According to the *stress/strain* model (Karasek, 1979; Spérandio, 1988; Schlegel, 1993; Raufaste et al., 2004), MWL includes two components: *stress* (task demands) and *strain* (consequences of stress on the individual). The relevance of this approach that emphasizes task demands remains evident in the current international standard on MWL (ISO, 2017), which also adopts the *stress/strain* model.

Furthermore, MWL is a relative concept considering it depends on the task's demands in relation to the amount of resources the operator is willing or able to allocate and process (Meijman and O'Hanlon, 1984; De Waard, 1996). In this context, “*able* to allocate” gains salience given that mental resources have a limited capacity. The Multiple Resource Theory (MRT) proposed by Wickens (1984, 1987, 2008) suggests that there exist multiple pools of attentional resources that reach a threshold when demands exceed the resource pool. Consequently, working memory, for instance, can only process a limited amount of information at any given time as argued by Information Theory (Shannon and Weaver, 1949).

This same principle also informs the Cognitive Load Theory (CLT; Sweller, 1988) which focuses on how intrinsic (IL-associated with the learning task itself), extraneous (EL- non-essential load mostly related to task instructions), and germane (GL-imposed by the learner's deliberate use of cognitive strategies for learning) loads impact the working memory of a learner (Sweller, 1988; Van Merriënboer and Sweller, 2010; Leppink et al., 2014; Young et al., 2015, 2021; Orru and Longo, 2019). Sweller (2010) re-conceptualized CLT by introducing the concept of element interactivity. *Elements* refer to the learning content that can be processed either independently in working memory (like a word list to memorize) or simultaneously (like elements in a mathematical equation). This determines the interactivity level between the elements. Germane learning is the extra effort required for learning (schema construction). However, for GL to be effective, sufficient working memory must be available. If not, EL can be reduced (such as by grouping words by meaning). In contrast, IL remains constant for a specific level of expertise. Therefore, GL is related to IL, which depends on the degree of interactivity of the task item, and to EL, which should not be high to make space for GL.

Mental workload and cognitive load are related concepts since they have common theoretical frameworks (such as the Memory model of Atkinson and Shiffrin, 1968; Information Theory, Shannon and Weaver, 1949) that are reflected through common measurement types such as performances and questionnaires. Moreover, some explanative dimensions investigated through questionnaires appear common between MWL and cognitive load models (Naismith et al., 2015). Thus, they show similar challenges when measured. Consequently, our discussion could also concern cognitive load. Yet, CLT has been developed to bring the explanatory model to a specific study domain, the learning one. In our study, we aim to decontextualize from any situation in order to adapt to multiple contexts. Mental workload, which is derived from several conceptual models that are not necessarily attached to an application domain, becomes therefore more appropriate. For this reason, this study is not on CLT, but on MWL assessment and prediction.

**Figure 1:**
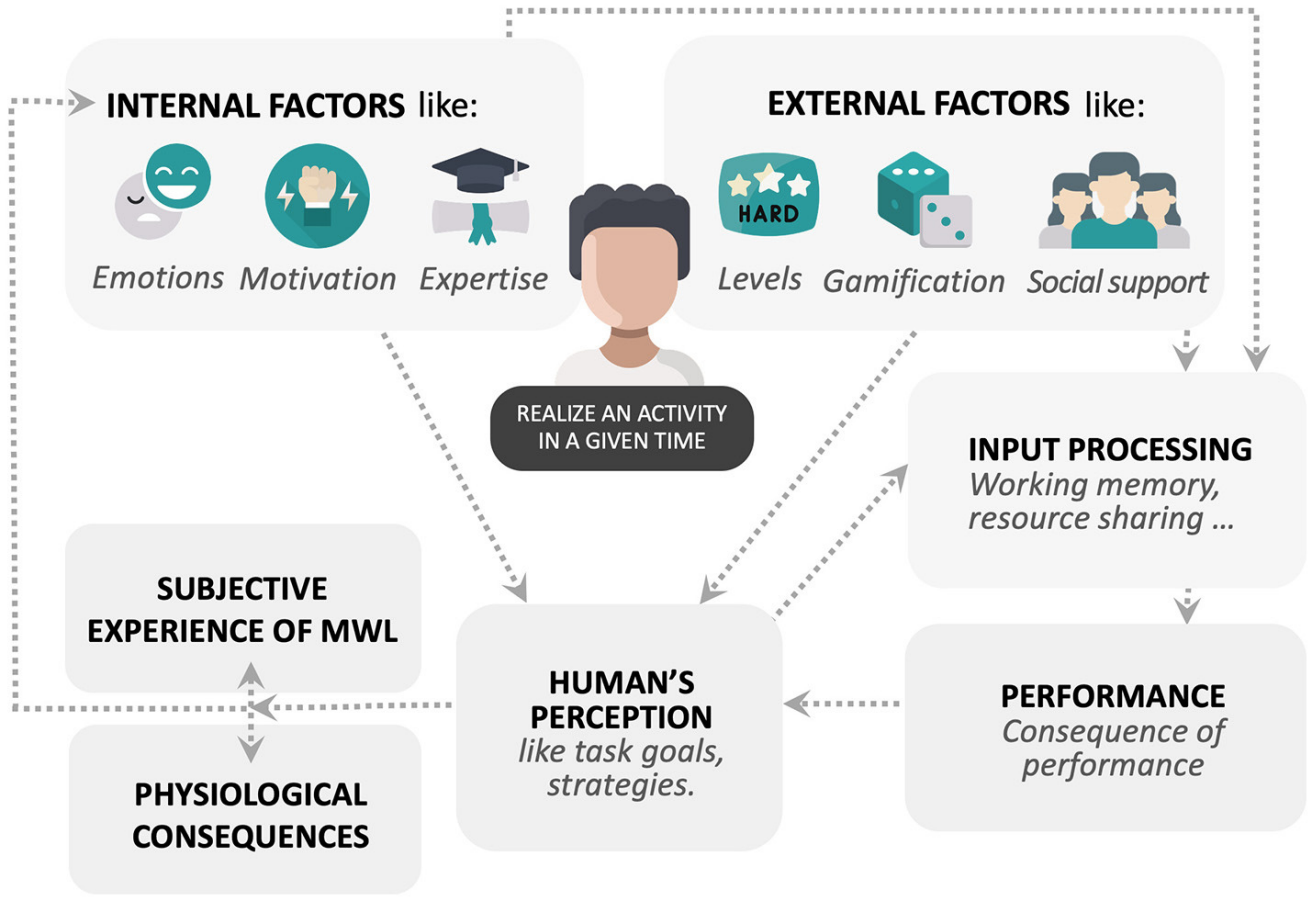
Conceptual framework of the mental workload factors.

The conceptual framework of MWL can be considered as follows (Figure 1): an individual is influenced by internal (endogenous) and external (exogenous) factors when she/he performs an activity. The inputs of this activity are then processed according to these factors, which can have an impact on performance. Some authors (Hart and Staveland, 1988; Spérandio, 1988; De Waard, 1996; Young et al., 2015) have already established a link between task demands, MWL, and performance. Performance influences an individual's perception of what she/he is expected to do, using strategies, effort, and expending resources to accomplish the task objectives (Hart and Staveland, 1988), and an individual's perception has an impact on subjective experience and physiological consequences (Wickens et al., 2015; Young et al., 2015). Therefore, we can observe a loop back with internal factors like subjective experience which can increase or decrease the motivation of the individual.

If an individual is faced with a situation that increases demands, she/he activates additional mental resources, which will result in higher levels of MWL (Dimitrakopoulos et al., 2017). Thereby the individual is able to shift from a given MWL level to a higher level. Several authors (De Waard, 1996; Martin, 2013; Young et al., 2015) have proposed three MWL levels. First, the mental underload, which according to Young et al. (2015), is an excessive stimulation leading to underload as resources are either allocated elsewhere or reduced by underuse. Second, an intermediate MWL is considered a “comfort zone” where performance is optimal (Dehais et al., 2020). Third, mental overload when the individual faces more stimuli than she/he can handle while preserving her/his own performance standards (Young et al., 2015).

Based on the definition proposed by Longo et al. (2022), which has emerged from hundreds of definitions, we considered mental workload as “*the degree of activation of a finite pool of resources, limited in capacity, while cognitively processing a primary task over time, mediated by external dynamic environmental and situational factors, as well as affected by definite internal characteristics of a human operator, for coping with static task demands, by devoted effort and attention.”* Therefore, for the purposes of this study, we adopted this MWL definition.

### Application areas of mental workload

**Figure 2:**
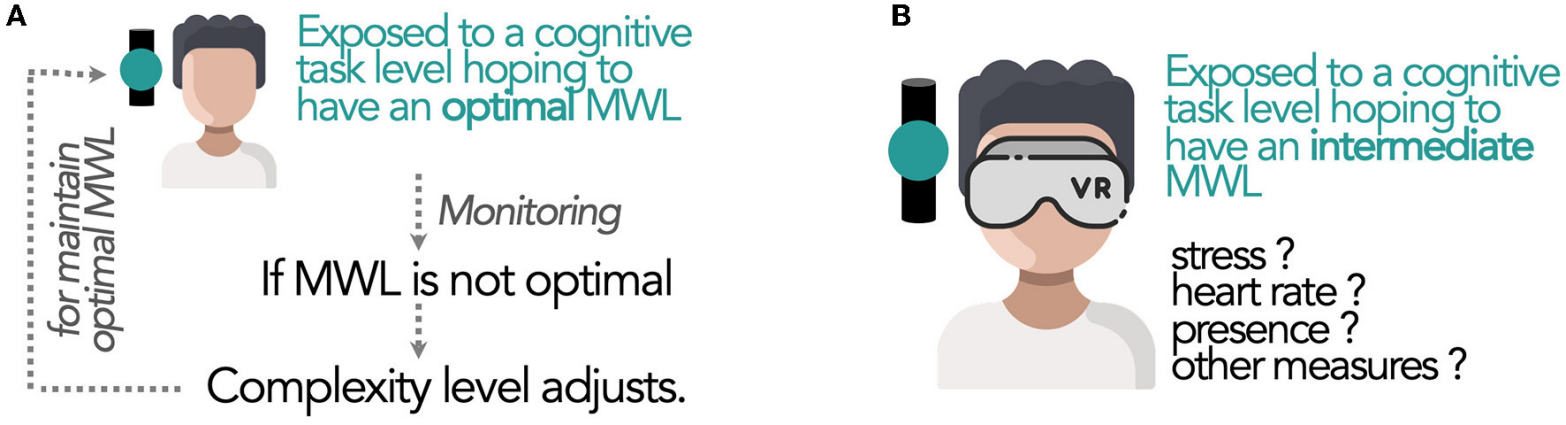
New possible use of the MWL concept in emerging applications such as medical remediation or Virtual Reality: **(A)** Online adjustment of task complexity to maintain optimized MWL or **(B)** assessment of the impact of a certain MWL level on different variables (such as stress or presence).

Due to its features, MWL is an indicator of the mental cost related to an activity. Consequently, it is often measured during the activity and/or after the activity. In recent times, MWL is mostly considered through operational situations such as automotive contexts (Milleville-Pennel and Charron, 2015; Foy and Chapman, 2018; Figalová et al., 2022) or aeronautic contexts like air traffic control (Averty et al., 2004; Mélan and Cascino, 2014; Li et al., 2022). Thus, MWL is usually measured as a consequence of the activity without any a priori certainty of the MWL value in which the person is when performing the task. However, in some clinical or research contexts, it would useful to be able to predict the MWL level before the activity. For example, in rehabilitation, technologies like Brain Computer Interface (BCI) allow following the cerebral activity during the achievement of cognitive tasks (Curran and Stokes, 2003; Carelli et al., 2017). In this case, the complexity level of the activity needs to be adapted to ensure the MWL is suitable for effective medical care. Thus, it is necessary to start with a known task in order to induce a certain MWL level, then, monitor the MWL level during the activity to adjust the characteristics of the task and keep the desired MWL level (Figure 2A).

In the research context, it also appears useful to maintain a certain MWL during an activity to measure its impact on different variables (such as stress or presence in Virtual Reality; Figure 2B). To achieve this, we need to identify MWL evolution, applying relatively close transitions between MWL levels. Thus, we aim to identify “sliding” levels of MWL. Moreover, MWL should be in the intermediate range as required for many contexts (like in Figure 2). Rehabilitation in a clinical domain is a concrete example since we do not try to underload or overload patients. On the contrary, we aim at intermediate levels of MWL so that patients can be engaged with positive feelings in the rehabilitation process. Nevertheless, such applications are not yet available since no task is identified to obtain distinct and closed MWL level intervals with certainty. Then, making these level classes of MWL become directly predictable from the complexity level could be a challenge while taking into account the availability of the real-time indicator. Thus, this study aims to propose several candidate cognitive tasks with distinct MWL levels that are in the intermediate zone. To identify the task(s) that best achieve distinct MWL levels, we propose to take advantage of the richness and complementary of different statistical methods.

### Requirements for these mental workload applications

Several conditions are necessary. At first, we must propose at least one task with different complexity levels taking into account that task difficulty is considered separately from task complexity. Indeed, task difficulty is a perceptual phenomenon while task complexity is considered an intrinsic property of the task (Longo et al., 2022). Thus, we will consider task complexity in order to set up cognitive tasks (CTs).

Secondly, the task should be context-free, that is, it should not depend on a specific operational situation (like car driving), but it should reflect the solicitation of the different cognitive functions more generically for it to be transferable and adaptable to various contexts. Several authors (Berka et al., 2007; Radüntz, 2017; Guan et al., 2021) have recommended studying MWL through CTs since real-life situations can be decomposed into several cognitive functions. Currently, several studies are mainly based on the N-back task (NBT) followed by the Sternberg task used to impact MWL during CTs. But these are two CTs, whose material is a set of letters of the Latin alphabet to be remembered, solicited only one type of cognitive function, the verbal working memory. However, our aim is to have several CTs soliciting several distinct cognitive functions. Furthermore, NBT and Sternberg are socially and culturally marked since people who cannot read and write or people using other alphabets (such as Cyrillic or Chinese characters) are excluded. Therefore, our goal is to have several CTs, which are independent of any context and possibly used by the greatest number of people.

Third, an important question for our study's purpose deals with the measure of MWL. There are different methods for assessing MWL which can be divided into three main categories (Miller, 2001; Galy et al., 2012; Muñoz-de-Escalona and Cañas, 2019): subjective, performance, and psychophysiological measures. The most relevant are the subjective category measures as they are the most valid and sensitive indicators (Hart and Staveland, 1988). Three multidimensional questionnaires are recognized for measuring MWL. These are the NASA Task Load Index (NASA-TLX; Hart and Staveland, 1988), the Subjective Workload Assessment Technique (SWAT; Reid and Nygren, 1988), and the Workload Profile (WP; Tsang and Velazquez, 1996). According to Rubio et al. (2004) and Paxion (2014), WP and NASA-TLX questionnaires are complementary as they compensate for the limitations of each other, and these questionnaires are based on different methodological or theoretical approaches. While the WP is founded on the Multiple Resource Theory (MRT) of Wickens (1984, 1987, 2008) cited above (Cf. *Conceptual framework of mental workload part*), the NASA-TLX is not rooted in a theoretical model, but in a methodological one. Following some twenty scientific studies, Hart and Staveland (1988) identified six factors used to determine the subjective MWL. Therefore, while one questionnaire (WP) is concerned with the saturation of the multiple pools of attentional resources, the other (NASA-TLX) is concerned with external (the first three items) and internal (the last three items) factors impacting MWL. All these elements are found in the definition of Longo et al. (2022) previously quoted.

Fourth, some authors point out that subjective measures can lead to biases since individuals may no longer remember the intrinsic details of the activity after performing it or respond in a way that satisfies assumed expectations (Spérandio, 1988; Cain, 2007). Nevertheless, subjective measures of MWL have a high diagnosticity criterion (ability to distinguish the source of the load) that is critical for the multidimensional concept of MWL (Wierwille and Eggemeier, 1993). Furthermore, subjective measures fulfill the validity criterion (the ability of the measure to assess what must be evaluated). However, subjective data have an important limitation for our purpose due to their lack of availability in real-time. Consequently, we also need accessible measurements during the activity taking into account the performance (such as reaction time or errors).

Fifth, MWL assessment methods based on performance analyses (primary performance or secondary performance during dual-task paradigm) assume that the performance obtained in the execution of one or more tasks will deteriorate if the demands expand (Cuvelier, 2012; Mandrick, 2013). Moreover, several MWL models and definitions establish a link between task demands, MWL, and performance (Cf. *MWL definitions part*). Nevertheless, performance measures might sometimes be limited to describe MWL given that operators may vary their effort to maintain a constant performance level (Reid and Nygren, 1988; Raufaste et al., 2004; Cain, 2007). Also, performance can be affected by other factors unrelated to workload such as stress or fatigue (Wickens et al., 2015). Hence, a problem occurs linked to the validity criterion. Performance measures, although less valid, might nevertheless be relevant because of the multiplicity of possible variations (such as completion time, errors, and correct answers), thus making the occurrence of a high-reliability criterion possible (capacity to detect modulations of MWL). In addition, among the MWL measurement criteria, performance satisfies the equipment criterion (the evaluation measure requires minimal equipment).

The last category of MWL measurement is psychophysiological measures. This term refers to the physiological response to psychological events. These measures are a natural type of workload index since work demands physiological activity (Young et al., 2015). The main advantages of this type of measure are their objectivity and the possibility to be gathered in real-time (Cain, 2007). For physiological measures, EEG is one of the most effective measures of MWL (Zhang et al., 2018). For instance, the study of Raufi and Longo (2022) demonstrated that EEG band ratios, specifically the alpha-to-theta and theta-to-alpha ratios could be treated as MWL indexes for the discrimination of self-reported MWL. However, for all psychophysiological measures, Kramer (1991) mentions the required equipment and technical expertise as disadvantages. Also, the possible occurrence of a noisy signal from physiological measurements (a weak signal-to-noise ratio) can lead to undesirable effects. Furthermore, physiological measures may involve different mental concepts such as stress (Katmah et al., 2021) or mental fatigue (Tran et al., 2020), and consequently, physiological data may be difficult to interpret and have a validity problem.

Therefore, obtaining a good indication of MWL in real-time will require having tasks with enough performance indicators. This will ensure a more reliable predictive model of MWL considering the richness and diversity of the performance dimensions. Thus, it could maximize the chances of having greater sensitivity, that is, the ability of the measure to discriminate changes in MWL. As a result, determining whether we can proceed without psychophysiological data whose benefit/constraint ratio is negative will become possible.

Finally, the MWL measure *via* questionnaires leads to huge variations between people (inter-individual). We can also observe this variation with a given person from one trial to another one, despite the same effective feeling (intra-individual). Thus, a good indicator of this variable must be able to predict, not only one value of MWL (the most likely for example) but a class of values that are susceptible to be observed in each condition. Our task must consequently be able to propose complexity levels inducing different classes of MWL in the intermediate zone of MWL while being sufficiently distinct to allow wide but not superposed classes of MWL values.

Thus, through this study, we aim to answer the following two questions:

1- Can we identify subjective mental workload classes corresponding to the complexity levels?
2- Can we predict these mental workload classes based on complexity levels and/or performance?

## Materials and methods

### Participants

Fifty-three healthy French-speaking participants, residing in France and meeting the inclusion criteria (fluent in French, right-handed, having a normal or corrected-to-normal vision, and normal or corrected-to-normal hearing), took part in this online experiment [28 women, 24 men, and 1 non-binary person corresponding to an “*identification with gender identities outside of male or female categories”* (Poirier et al., 2019)]. With an average age of 28.64 years (SD = 6.89 years), the voluntary participants were told the real purpose of the experience. Approximately 96.23% of them (51 people) had at least a bachelor's level + 2 years of education including 43.4% with a master's degree. The rest of the participants had a bachelor's level of education. Moreover, 66.26% (33 people) worked for Onepoint company, 9.43% were from the University of Nantes, and the others were acquaintances of the experimenters.

### Tasks and procedure

To address the challenges of having many participants in a given time despite the pandemic lockdown, we opted for an online study. Therefore, instructions for the test were designed for an optimal remote experiment (such as the need to sit in a quiet room or to put the phone on mute).

#### Cognitive tasks

All the tasks were created using *Unity3D* software (Version 2019.4.30f1).

**Figure 3:**
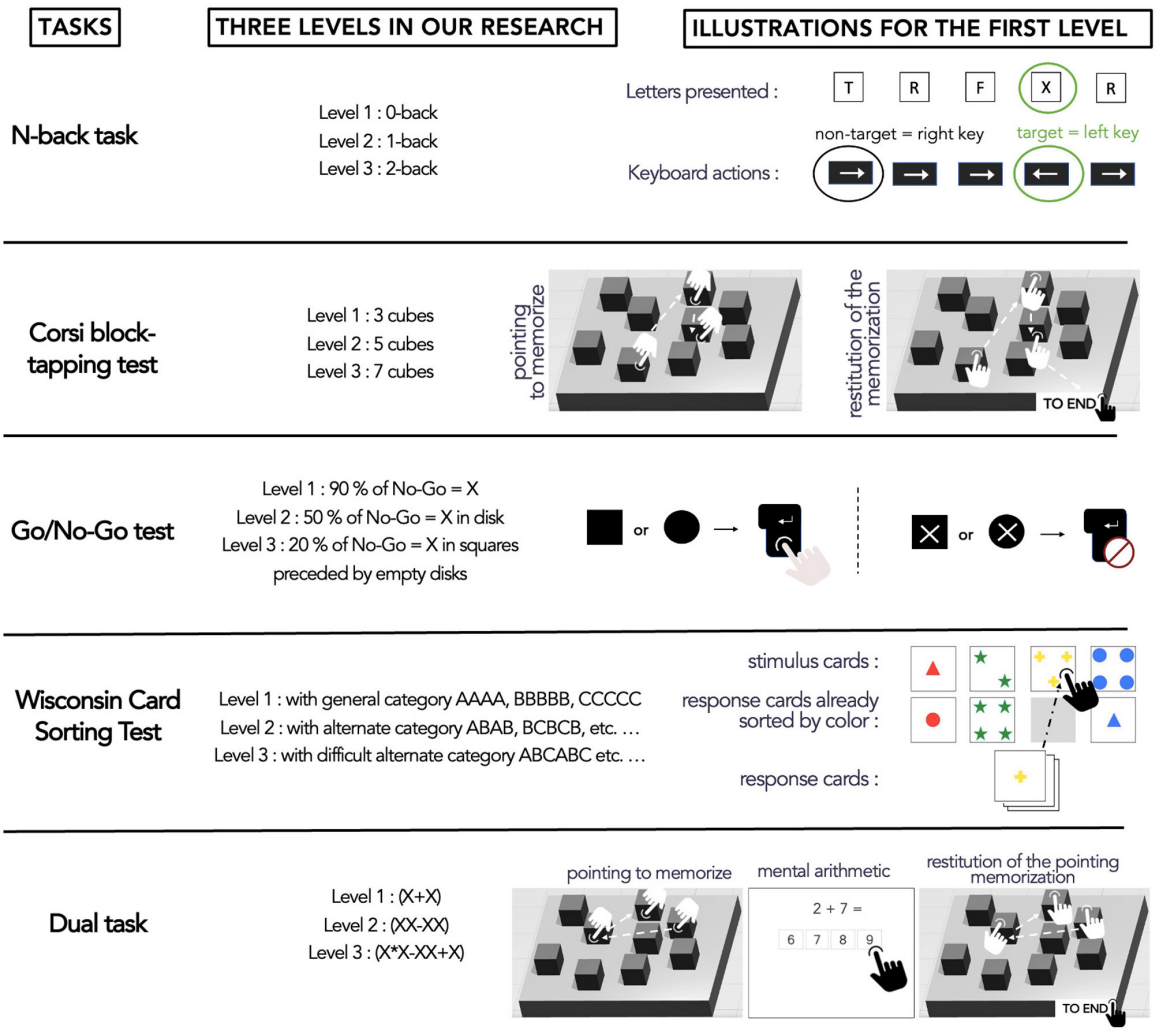
Experimental design of the study where participants were randomly assigned tasks.

Participants undertook five cognitive digitized tasks on their personal computers. We had selected beforehand popular cognitive tasks whose structure made it possible to induce several dimensions. The tasks were (Figure 3): N-back task (NBT), Corsi block-tapping test, Wisconsin Card Sorting test (WCST), Go/No-Go (GNG) test, and a Dual task (DT). All participants were exposed to tasks in random order. The NBT is renowned to have complexity levels inducing MWL levels in the intermediate zone of MWL corresponding to low MWL, medium MWL, and high MWL for respectively 0-back, 1-back, and 2-back levels (Arvaneh et al., 2015; Dimitrakopoulos et al., 2017; Ries et al., 2018). Thus, NBT served as a benchmark to calibrate the complexity levels of the other tasks during pre-tests where we could evaluate several levels with at least five participants. Performance measures served as indicators for selecting the three complexity levels. However, with these measures, we could not obtain comparable indicators as they were different from task to task and not easily transferable (Sirevaag et al., 1993; Raufaste et al., 2004). We, therefore, defined a common performance indicator for all tasks, corresponding to the percentage of *Expected responses*. For example, if the participant responded properly to all her/his questions, the *Expected responses* were 100%. Thus, during pre-tests with several complexity levels, we selected three of them according to their *Expected responses*.

##### N-back task

N-back task (NBT) is a verbal memory span test that involves the refreshment capacity of working memory proposed by Kirchner (1958). The test requires participants to react when a stimulus (like a letter) is the same as the stimulus presented before it (if it occurs, it is a target). In our study, the letters were presented centrally for 500 ms each, followed by a 1,500 ms interstimulus interval. Participants were invited to respond by indicating whether the letter was a target (pressing the left directional key) or a non-target (pressing the right directional key). Three complexity levels (0-back; 1-back; 2-back) were proposed with 20 trials (including 6 to 8 target letters) for each level. For the 0-back level, the target was the letter “X”. For the 1-back and 2-back levels, the target was the letter presented immediately or two letters before the current one. Finally, performance measures had six dimensions: *No answers, False alarms* (pressing on the target key when a non-target letter was displayed), *Omissions* (not pressing on the target key when it was a target letter), *Reaction time for all items, Reaction time for correct answers*, and *Expected responses*.

##### Corsi block-tapping test

Block-tapping task or Corsi test (Corsi, 1972) is a visual-spatial memory span task that consists in remembering a sequence of cubes and reproducing the sequence in the same order. The material was the same as in the study by Kessels et al. (2000). Three complexity levels (3 cubes, 5 cubes, and 7 cubes) were proposed with two sequences each. As such, the maximum number of items varied from one level to another. In this case, each performance data was expressed as a percentage (divided by the maximum possible number) aiming to compare the different complexity levels. Finally, performance measures had seven dimensions: *Pointed cubes, Correct cubes, False cubes, Omissions*, (not pressing a cube), *Exact sequences* (number of cubes pointed in the expected order and position), *Total time*, and *Expected responses*.

##### Go/No-Go

The Go/No-Go task (GNG) is a mental inhibition of the motor response task. For our study, we based it on the GNG version of Vidal et al. (2012). The stimuli were presented in a randomized manner and consisted of black disks and black squares on a white background. A shape appeared either full or empty. In the first level, when the shape was empty, individuals had to click on the ENTER key of the keyboard with their right hand (Go target). Conversely, when the letter X appeared randomly on the shapes, individuals had to inhibit their motor response. The X was therefore a No-Go target. Three complexity levels were proposed. A simple level (with 90% of No-Go = X), an intermediate level (with 50% of No-Go = “X” in disk), and a difficult level (with 20% of No-Go = “X” in squares preceded by empty disks). Each level had 10 items presented for 200 ms followed by a fixation point of 500 ms. Performance measures had five dimensions: *False alarms* (pressing the key when a No-Go target was displayed), *Omissions* (not pressing the key when it was a Go target), *Reaction time for correct answers, Total time*, and *Expected responses*. Also, *False alarms* and *Omissions* were expressed as a percentage (divided by the maximum possible number) to compare the different complexity levels. The maximum number of items varied from one level to another. For example, in 10 items, level 1 could express a maximum of 9 *False alarms* (since this level had 9 No-Go), level 2 could express a maximum of 5 *False alarms* (since this level had 5 No-Go) and level 3 could express maximum 2 *False alarms* (since this level had 2 No-Go).

##### Wisconsin card sorting test

The WCST (Heaton, 1981) is a card-sorting task during which the participant must follow a classification rule and adapt to various rule changes. Therefore, this task enables the involvement of mental flexibility, which represents the ability to shift between tasks (Miyake et al., 2000). For the present study, we were guided by the French version validated by Godefroy and GREFEX (2008) dealing with the Modified Card Sorting Test (Nelson, 1976). The top of the computer screen displayed four target cards (one with a red triangle, one with two green stars, one with three yellow crosses, and one with four blue circles). At the bottom of the screen, there was a stack of several cards named response cards which were scrolled every 2,000 ms. In this stack, the shapes could be a circle, cross, star, or square, the numbers could be 1, 2, 3, or 4, and the colors yellow, red, green, or blue. Three complexity levels were proposed: Level 1—sorting by general dimensions (type of figure, color of the figure, or the number of figures). Participants had to focus on a single dimension to sort the cards. Level 2—sorting by alternating dimensions. Participants had to alternate between two distinct dimensions (for example color and number of figures) on each trial. Level 3—sorting by complex alternating dimensions. Participants had to alternate between the three dimensions in each trial. The performance measures had six dimensions: *Number of errors, Perseverative errors* (when the incorrect answer matched the category used by the person for his/her previous answer), *Reaction time for all items, Reaction time for perseverative errors, Total time*, and *Expected responses*.

##### Dual task

This task was partly based on the previously mentioned Corsi test. But the Corsi part of this Dual task still had three cubes to be memorized. In parallel, a mental calculation task was added to disturb the memorization process. Consequently, this task was inserted between the pointing of the cubes by the experimenter (thus the beginning of the memorization) and the restitution of the pointing by the participants. The calculation task was chosen since we needed a Dual task with visual-spatial memory and verbal memory. For this last task, it was necessary to have a fast and adjustable test at the complexity level. Therefore, we used a calculation task based on So et al. (2017) study, with four clickable choices of possible answers. Three complexity levels were proposed with two sequences each. A simple level with single-digit addition, an intermediate level with double-digit subtraction, and a difficult level with mixed arithmetic operations including multiplication and subtraction. Finally, performance measures offered nine dimensions, including the same performance dimensions as the Corsi test in addition to mental calculation performance corresponding to *Correct answers* and *Calculation total time*.

#### Questionnaires

As mentioned in the introduction, as NASA-TLX and WP questionnaires are multidimensional and complementary, they were used in the present study.

##### NASA-TLX questionnaire

The NASA-TLX (Hart and Staveland, 1988) rates perceive workload on six different sub-scales*: three* dimensions associated with the activity (mental demands, physical demands, time pressure), two dimensions associated with the strategies (performance, effort), and one dimension specific to the individual's emotional state (frustration). After each task level, the participant must score each dimension from 0 (no demand) to 100 (maximum demand). In our study, we considered each dimension of the NASA-TLX questionnaire and its overall score. For the last one, we used the unweighted version of the questionnaire by averaging the six dimensions to calculate a Raw Task Load Index (RTLX). This method was validated by Byers et al. (1989). Thirty years later, Cegarra and Morgado (2009) demonstrated that the French version showed a strong correlation between the weighted score (TLX) and the unweighted score (RTLX).

##### Workload profile questionnaire

The WP questionnaire (Tsang and Velazquez, 1996) was based on the Multiple Resource Theory (MRT) of Wickens (1984, 1987) and asked the participants to provide the proportion of attentional resources used after they had experienced the tasks. Thus, the workload dimensions used in this technique were defined by the resource dimensions hypothesized in the MRT: *Perceptual central processing, Response selection and execution, Spatial processing, Verbal processing, Visual processing, Auditory processing, Manual output*, and *Speech output* (Rubio et al., 2004). For each task, the participant had to provide a number between 0 (no demand) and 100 (maximum demand) representing the proportion of attentional resources used in each of the eight workload dimensions.

Since there were no solving, selection, or auditory and speech tasks in our study, only four of the eight dimensions were analyzed. Indeed, referring to the main article of the questionnaire (Tsang and Velazquez, 1996, p. 362), this one can only be rated in a one-dimensional way. Thus, for our study, we considered the *Spatial processing scale* (WP3), *Verbal processing* (WP4), *Visual processing* (WP5), and *Manual output* (WP7).

#### Experimental procedure

Each participant was clearly informed about the objectives and the course of the study before signing the consent letter to participate in the online study. This experiment adopted a 5 × 3 within-subject design with 5 task types and 3 complexity levels in tasks. The experimental phase proceeded as follows:

- Presentation of a summary of the study by email or through professional social networks.
- When the person showed interest, the experimenter sent her/him a link with a personal password.
- Therefore, the participant was connected to the online experimental session once the password was entered, and an electronic signature on the consent letter was requested before the pre-test questionnaire (questions about age, gender, and level of education).
- Once these phases were completed, they performed the CTs in two 1-h sessions. All tasks included instructions on how to undertake the tasks (in the form of text, images, and videos) followed by a training session to become familiar with the presentation of the stimuli and the required interactions. The total duration of one task was about 15–20 min.
- After each task level, an electronic version of the NASA-TLX and WP questionnaires was delivered.

All participants were exposed to tasks and complexity levels in random order. At the end of the study, all participants received 15 euros in vouchers.

#### Data analysis

To answer the two research questions, the following research hypotheses were defined.

Concerning the question “*Can we identify subjective MWL classes corresponding to the complexity levels?*”

**H1**: If the complexity levels lead to distinct MWL classes, then the means for each complexity level will be different and the MWL data will probably be spontaneously grouped according to their proximity into MWL value classes, and these classes will potentially be correlated to those obtained *via* the complexity levels.

 → First, for all subjective measures (NASA-TLX and WP questionnaires), we normalized these data through the correction proposed by Cousineau (Morey, 2008). This normalization was based on within-subjects confidence intervals which were recommended for subjective data like those from questionnaires. Cousineau's method could be described as follows. Let *y*_*ij*_ be the *i*th participant's score in the *j*th condition (*i* = 1, ..., *N*; *j* = 1, …, M). Then, the normalized observations *z*_*ij*_were defined as follows:


$$\begin{array}{l} z_{ij}=y_{ij}-\frac{1}{N}\;\overset{M}{\underset{j=1}{\sum }}y_{ij}\;+\frac{1}{NM}\overset{N}{\underset{i=1}{\sum }}\overset{M}{\underset{j=1}{\sum }}y_{ij} \end{array}$$

In our case, we subtracted the mean of all conditions (the five tasks) and added the mean of every participant in all experimental conditions.

 → Then, we carried out ANOVAs to compare the MWL class averages in relation to the complexity levels. For each specific analysis, the characteristics of the ANOVAs will be specified in the result section.

 → Subsequently, we performed K-means classification to identify the “spontaneous” MWL classes obtained with our protocol. We believed comparing MWL averages through a grouping based on the complexity levels could artificially suggest the presence of different MWL averages by hiding the overlapping of classes. We, therefore, used another method to observe the groupings of MWL values, not based on the complexity level but on the proximity between the observed values. The classes obtained were then compared to those observed by considering the complexity levels.

Therefore, the other step in analyzing our results was to investigate if we could obtain three MWL classes without considering the complexity level. For this purpose, we used the K-means technique to perform clustering. Existing studies (Al-Mohair et al., 2015; Shaheen et al., 2020) employing clustering referred to K-means as a simple, reliable, and robust technique for clustering. K-means clustering divided the data into K sets. The number of possible sets depended on the nature of the data or the plausible possibility of the number of sets that a data could offer.

 → For all CTs, we kept the whole set of dimensions of the NASA-TLX, since this questionnaire must be considered in its entirety (Hart and Staveland, 1988) unlike the WP questionnaire. Indeed, referring to the main article of this questionnaire (Tsang and Velazquez, 1996, p. 362), WP is specified to be rated in a one-dimensional way. Therefore, for each CT and before the clustering step, we identified the dimensions of the most relevant WP depending on the logic of the task, that is, the cognitive demands it generated. We also considered the results of the previous analysis (ANOVAs) showing the most affected WP dimension by each task. It is worth noting that in this study, we specifically selected the dimensions of the WP questionnaire that we deemed relevant for examination. However, with this protocol, we did not measure all the resources spent (8 dimensions in total). Consequently, WP results were limited to the presented tasks and could not be generalized across other tasks.

 → Finally, we calculated the correlation between MWL classes obtained from the K-means with the classes obtained *via* the complexity levels.

Concerning the question “*Can we predict these MWL classes based on complexity levels and/or performance?*”

**H2**: If the performance measures are rich and diversified, then it will be likely to find a model which will correctly classify the MWL according to the complexity levels and/or performance.

For this research question, we wanted to predict belongingness to a class of MWL values. For this purpose, a supervised classification (specific to each CT) was used to classify observations in different categories of the dependent variables, aimed at identifying whether an individual stayed in an MWL class or changed MWL class over time.

 → It is interesting to note that the variable to be predicted (subjective MWL) corresponded to the classes established by K-means. For each analysis, we first considered complexity level as a variable for the model, followed by the comparison of the model precision with the one obtained when the complexity level was removed from the model and only performance was considered by the model.

 → Concerning the supervised classification technique, we selected Linear Discriminant Analysis (LDA). Besides its low computational cost (compared to the Support Vector Machine—SVM for instance), the LDA has been used in the domain of cognitive tasks (Abibullaev and An, 2012; Yoo et al., 2020). Moreover, the hyperplane of LDA used to separate different classes allowed us to identify the features that maximize the between-class variances, while minimizing the within-class variance (Mohanavelu et al., 2022). This was one of the aims of our study.

The LDA model in this study calculated the balanced accuracy for our case of multiclass analysis (ratio of true positives to total positives). We then considered the cross-validation score obtained with complexity levels and performance combinations and the one obtained with only performance combinations. The details of performance measures for each task were described in the methods section. Concerning the selection of MWL variables, we used the best selection for each task based on previous results (ANOVAs, K-means, and correlation coefficient between MWL classes with K-means and MWL classes with complexity levels).

 → We then calculated the importance of the different variables for the model. For this, we used the Permutation Feature Importance (PFI) method. PFI is defined as the decrease of the model score when the value of a predictor variable is randomly shuffled. PFI permutes the features at each round, then removes a feature from the list and associates the increase in error as the rank to the feature previously removed. Unfortunately, PFI showed a significant inconvenience as it did not indicate how many features to use but only the most highly ranked (Bel, 2020).

**Figure 4:**
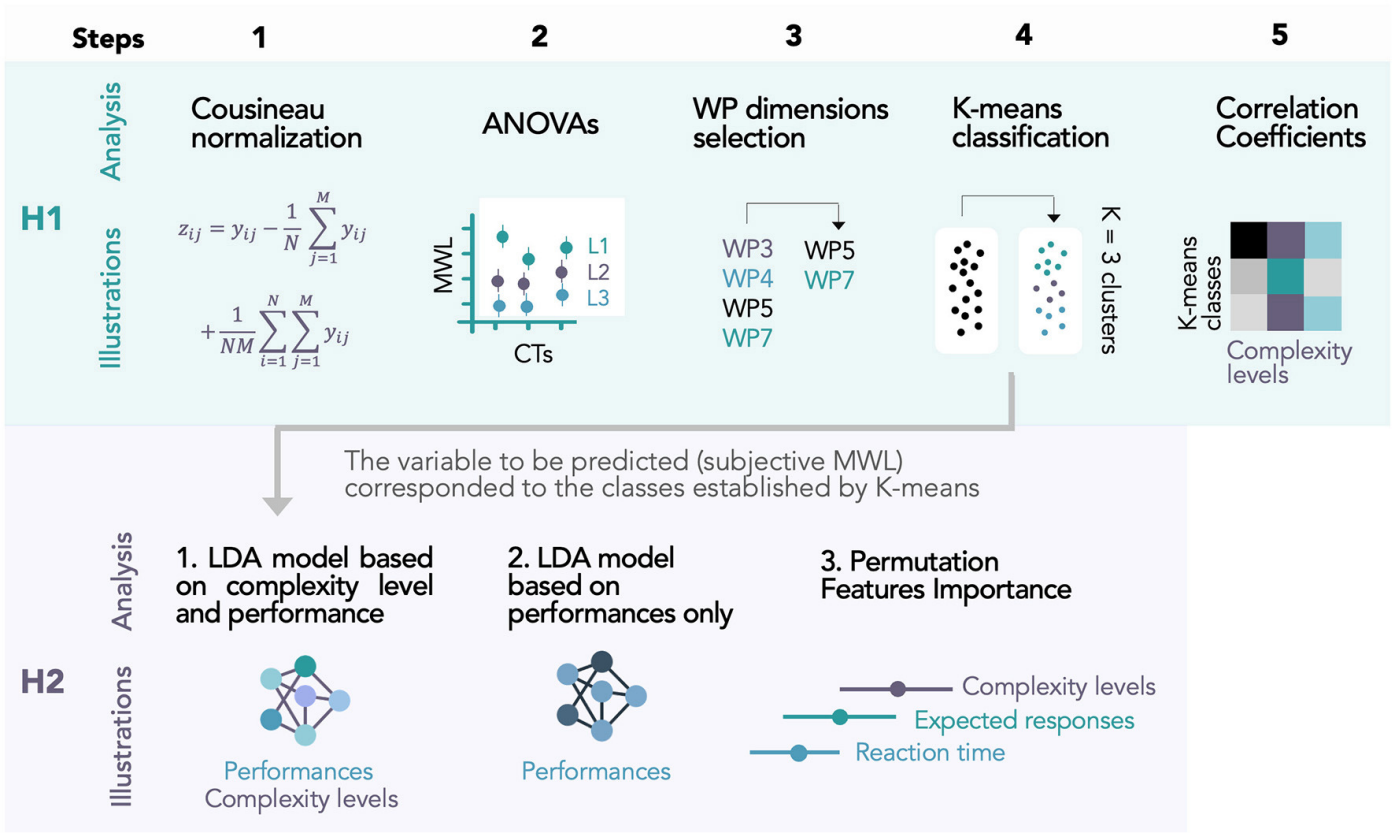
Diagram with all the steps of data analysis for the two research hypotheses.

Finally, to have an overall view of our analysis procedure, we proposed a diagram (Figure 4) with all the steps of data analysis for the two research hypotheses.

## Results

We started the analysis of our results by making sure that the complexity levels of our five CTs generated three distinct levels of performance.

### Complexity level and performance

To compare the performances between tasks, we established a performance criterion common to all five CTs, the *Expected responses* (Cf. *Cognitive tasks* part). Moreover, some distributions of our data were not normal, and variances were not homogeneous. For this reason, we used non-parametric statistics with the *JASP* software (version JASP 0.16). A non-parametric Repeated-Measures ANOVA (with the Friedman test) was performed (with complexity level as the independent variable and performance as a dependent variable) followed by Wilcoxon signed-rank tests and Conover's *post hoc* comparisons.

**Figure 5:**
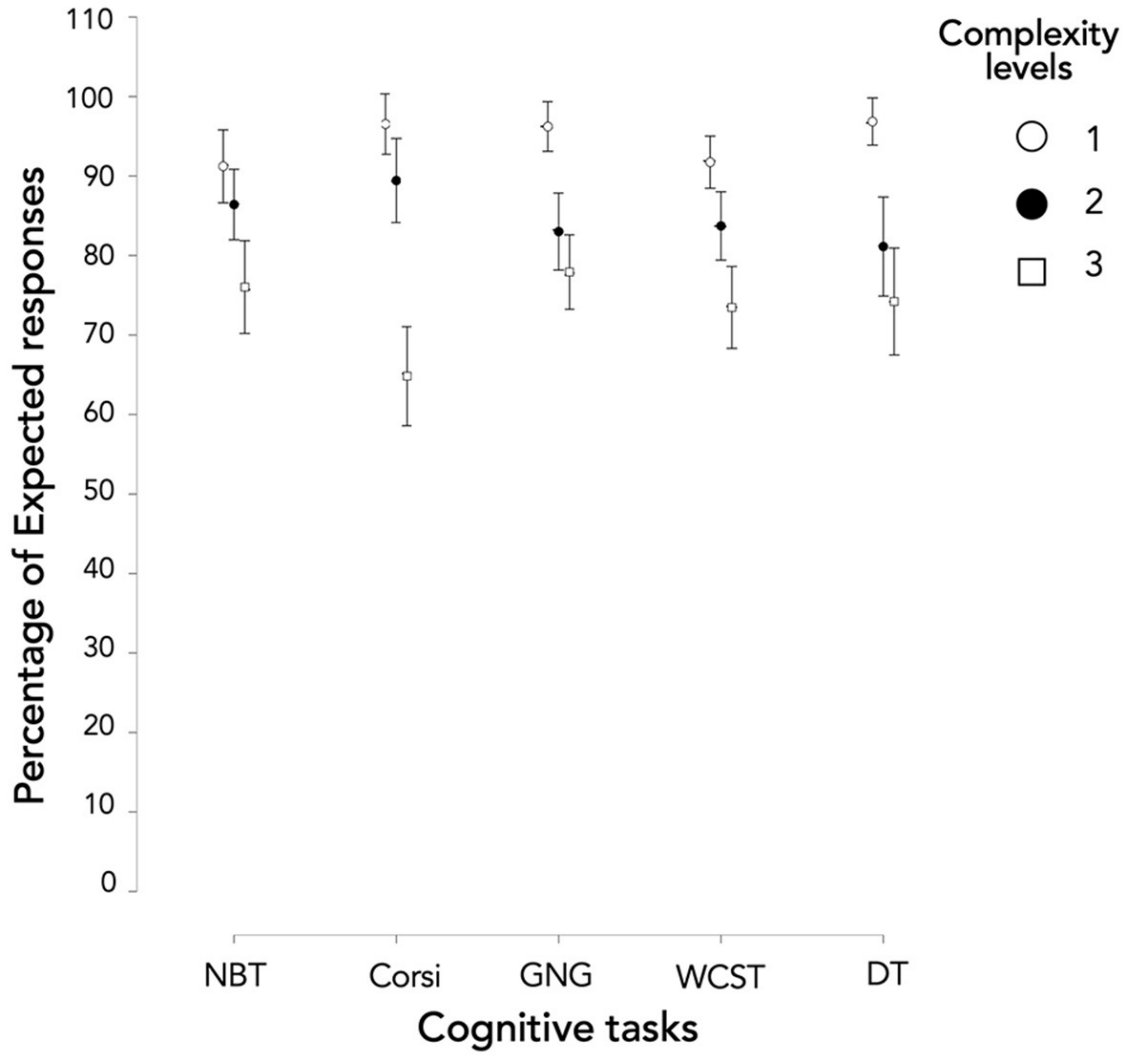
Performance in terms of Expected responses according to the cognitive tasks and the levels.

Concerning *Expected responses* (Figure 5), we could observe a main effect of complexity level independently of the tasks (Supplementary Table 1A; *X*^2^(2) = 145.255; *p* < 0.001). Levels 1 and 2 were significantly different (Supplementary Table 1B;*T*_(740)_ = 2.887; *p* = 0.004), as well as levels 2 and 3 [*T*_(740)_ = 2.592; *p* = 0.010]. Complexity level 1 was the least complex level and level 3 proved to be the most complex.

Moreover, considered task by task, all complexity levels were significantly different (Supplementary Table 1C) except levels 2 and 3 of GNG (*W* = 561.000; *p* = 0.086) and DT (*W* = 460.000; *p* = 0.102). Consequently, the three complexity levels generated three distinct levels of *Expected responses* for NBT, Corsi, and WCST. However, levels 2 and 3 did not show a significant difference when using GNG and DT.

### Correspondence between MWL classes and complexity levels

For this part, non-parametric statistics with the *JASP* software (version JASP 0.16) were used.

A non-parametric Repeated-Measures ANOVA (with the Friedman test) was performed with complexity levels as the independent variable and subjective measures (NASA-TLX and WP) as the dependent variable. Then, Wilcoxon signed-rank tests and Conover's *post hoc* comparisons were used.

### NASA-TLX questionnaire

**Figure 6:**
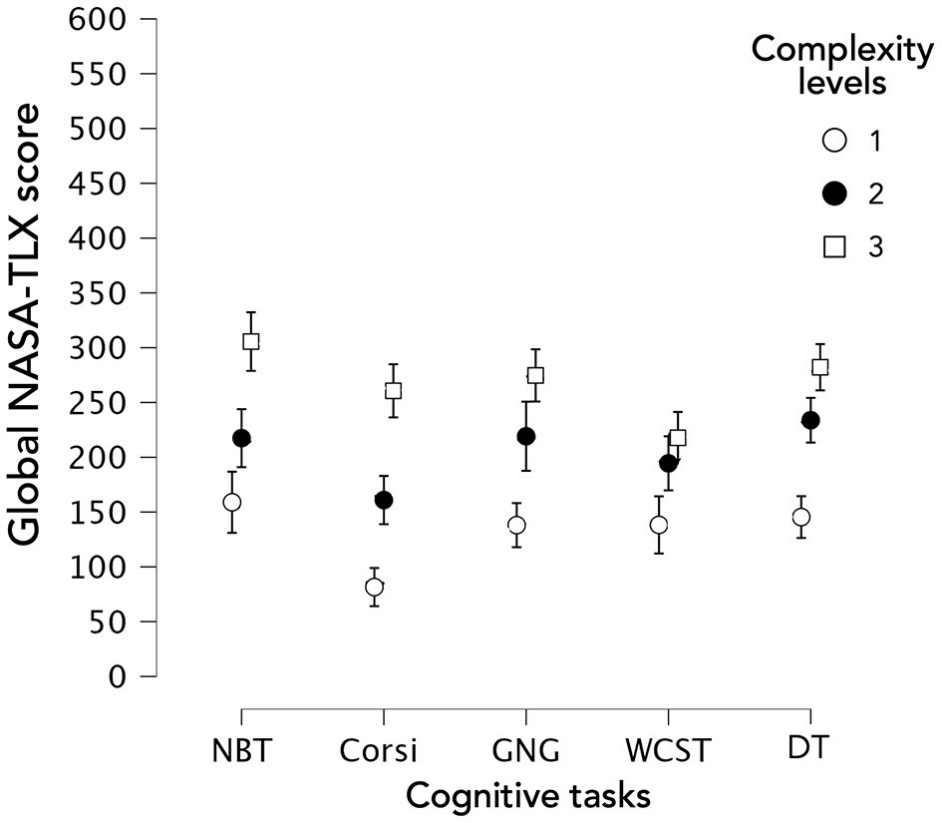
Mental workload based on overall NASA-TLX score according to the type of task and the complexity level.

Concerning the *overall NASA-TLX score* (corresponding to the sum of the six NASA-TLX dimensions, Figure 6), a significant main effect of complexity level occurred (Supplementary Table 2A; [*X*^2^(2) = 179,626; *p* < 0.001]. Independently of the task, all complexity levels were significantly different (Supplementary Table 2B). Furthermore, considered task by task, all complexity levels were also significantly different (Supplementary Table 2C). Hence, the three complexity levels generated three distinct levels of overall MWL.

Moreover, considered level by level, NBT and GNG did not turn out significantly different for the three complexity levels. Besides, this could also be observed between NBT and DT, and GNG and DT (Supplementary Table 2D).

**Figure 7:**
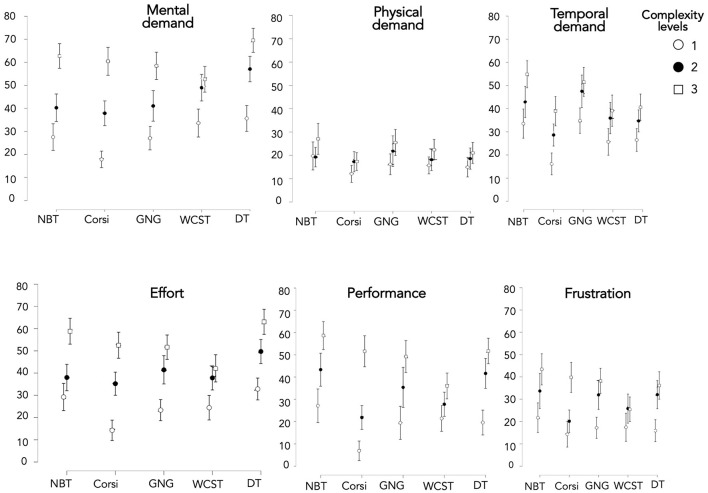
Scores of the 6 dimensions of NASA-TLX depending on the task and complexity level.

Concerning the dimensions score of the NASA-TLX questionnaire, in *Mental demand* (Figure 7) a significant main effect of complexity level occurred [Supplementary Table 3; *X*^2^(2) = 164,416; *p* < 0.001]. Independently of the task, all complexity levels were significantly different. Moreover, considered task by task, all complexity levels were significantly different except for levels 2 and 3 of WCST (*W* = 546.000; *p* = 0.135). Thus, the three complexity levels generated three distinct levels of *Mental demand* except for WCST.

Concerning *Physical demand* (Figure 7), MWL based on *Physical demand* turned out to be the lowest for all the tasks considered when compared to the other dimensions, with an average of below 30 in 100 on the Likert scale. Furthermore, despite a significant main effect of complexity level [Supplementary Table 3; *X*^2^(2) = 14.769; *p* < 0.001], independently of the task, the complexity levels were not significantly different. This could be explained by the effect size—Kendall's coefficient of concordance (Kendall's *W*)—used for assessing agreement among raters. Kendall's *W* ranged from 0 (no agreement) to 1 (complete agreement). Concerning the main effect of the complexity level for *Physical demand*, it turned out to be relatively small (*W* = 0.061). Moreover, when considered task by task, the three complexity levels were not significantly different. Hence, our three complexity levels did not generate three distinct levels of *Physical demand*.

With regard to *Temporal demand*, a significant main effect of complexity level occurred [Supplementary Table 3; *X*^2^(2) = 49.422; *p* < 0.001]. Independently of the task, only levels 1 and 2 [*T*_(740)_ = 2.008; *p* = 0.045] were significantly different, as well as levels 1 and 3 [*T*_(740)_ = 3,286; *p* = 0.001]. However, considered task by task, all complexity levels were significantly different except for levels 2 and 3 of GNG (*W* = 419.000; *p* = 0.689). Thus, our three complexity levels generated three distinct levels of *Temporal demand* except for GNG.

Concerning *Effort*, a significant main effect of complexity level on all tasks occurred [Supplementary Table 3; *X*^2^(2) = 143.925; *p* < 0.001]. Independently of the task, all levels were significantly different. Moreover, considered task by task, all complexity levels were significantly different except for levels 2 and 3 of WCST (W = 518.000; *p* = 0.081). Hence, the three complexity levels generated three distinct levels of *Effort* except for WCST.

Concerning *Performance*, a significant main effect of complexity level on all tasks occurred [Supplementary Table 3; *X*^2^(2) = 137.616; *p* < 0.001]. Independently of the task, all levels were significantly different. When considered task by task, all complexity levels were significantly different, even for levels 2 and 3 of WCST (*W* = 409.500*; p* = 0.028). Thus, the three complexity levels generated three distinct levels of *Performance*.

Finally, with regard to *Frustration*, a significant main effect of complexity level on all tasks occurred [Supplementary Table 3; *X*^2^(2) = 81.364; *p* < 0.001]. Independently of the task, all levels were significantly different except for levels 2 and 3 [*T*_(740)_ = 1.662; *p* = 0.097] and when considered task by task, pairwise comparisons showed that it only concerned Corsi and DT.

To summarize, three complexity levels generated three distinct levels of *overall MWL*. This was the case for the *Performance* of all CTs. It also applied to *Mental demand* and *Effort* of all CTs except for WCST. Likewise, three complexity levels generated three distinct levels of *Temporal demand* except for GNG. Concerning *Frustration*, the results were different according to the CT and only Corsi and DT induced different *Frustration* levels as a function of complexity level. Finally, *Physical demand* was the only dimension that was not influenced by the complexity level whatever the CT and compared to other dimensions, MWL from *Physical demand* was the lowest for all the tasks considered.

### WP questionnaire

For this part, we detailed the results of each WP dimension that we had previously selected, namely WP3-*Spatial processing*, WP4-*Verbal processing*, WP5-*Visual processing*, and WP7-*Manual output* (Cf. *Workload Profile questionnaire* part).

**Figure 8:**
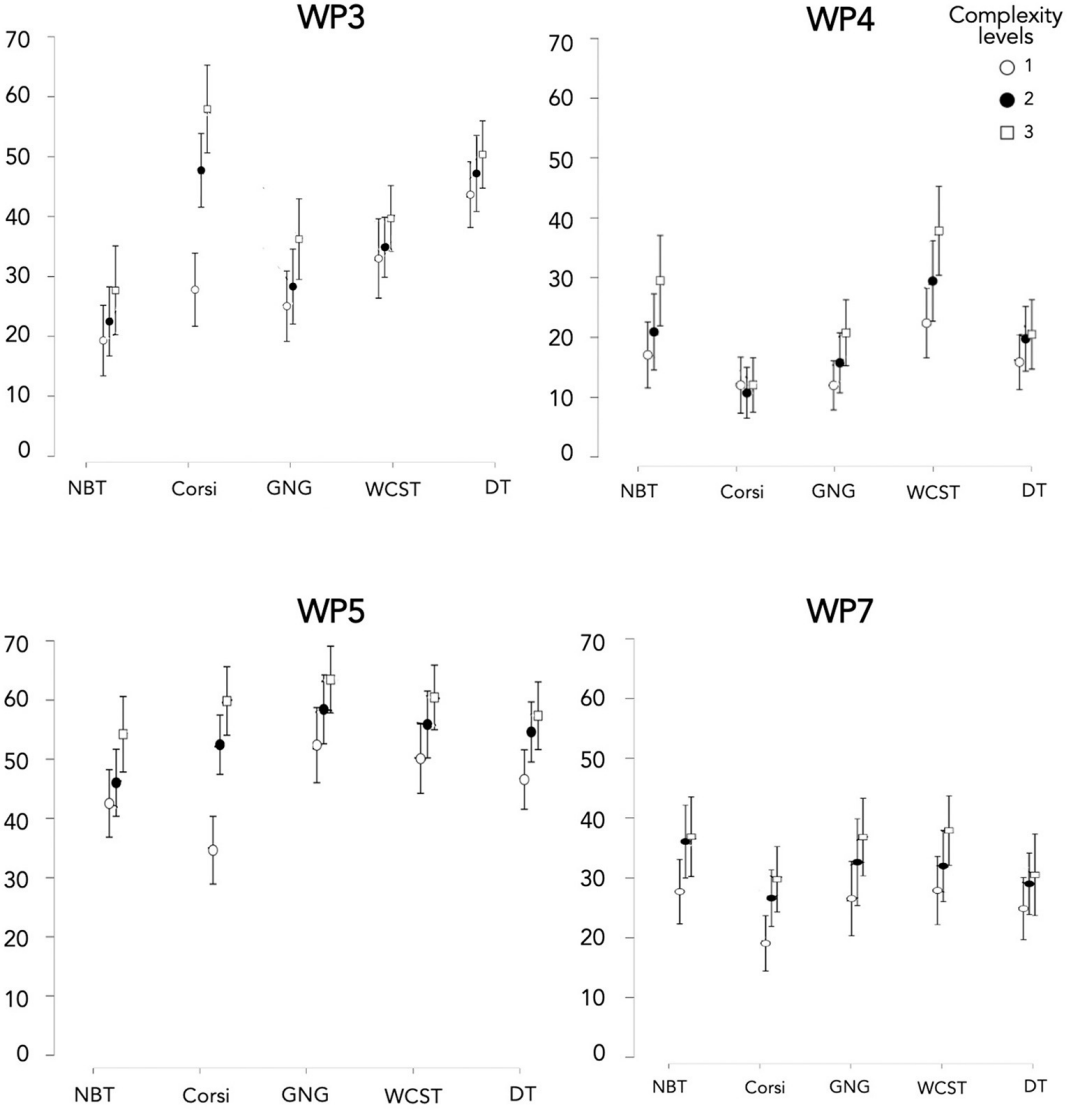
Mental workload score of four dimensions of the WP questionnaire (WP3, WP4, WP5, and WP7) depending on the task and complexity level.

Concerning WP5 (Figure 8), we could observe that compared to the other dimensions, MWL was the highest for nearly all the tasks considered, with an average of above 30 in 100 on the Likert scale. Furthermore**, **despite a significant main effect of the task [*X*^2^(4) = 13.893; *p* = 0.008], Conover's *post hoc* comparisons between tasks indicated no significant differences (Supplementary Table 4C). This could be explained by the relatively small effect size (*W* = 0.026). Moreover, a significant main effect of complexity level occurred on all tasks [*X*^2^*(2)* = 43.682; *p* < 0.001] but only levels 1 and 3 [*T*_(740)_ = 3.056; *p* = 0.002] were significantly different. Finally, when considered task by task, all complexity levels turned out significantly different only in NBT and Corsi.

Concerning WP3 (Figure 8), a significant main effect of the task occurred [Supplementary Table 4A; *X*^2^(4) = 86.422; *p* < 0.001]. The WP3 for DT and Corsi was significantly greater than for GNG. Furthermore, WP3 for NBT was significantly lower than others for all the tasks (except GNG). Moreover, a main effect of complexity level occurred [Supplementary Table 4A; *X*^2^(2) = 27.797; *p* < 0.001] but only levels 1 and 3 were significantly different [*T*_(740)_ = 2.447; *p* = 0.015]. Considered task by task, all complexity levels turned out significantly different for Corsi alone.

Concerning WP4, a significant main effect of the task occurred [Supplementary Table 4; *X*^2^(4) = 55.564; *p* < 0.001]. WP4 of NBT and WCST generated the highest MWL compared to the other CTs. Moreover, despite a significant main effect of complexity level [*X*^2^(2) = 13.809; *p* = 0.001], no level was significantly different from another, even for levels 1 and 3 [*T*_(740)_ = 1.699; *p* = 0.090]. Thus, when considered task by task, all complexity levels were significantly different for WCST alone.

Concerning WP7, the main effect of the task did not turn out statistically significant [*X*^2^(4) = 5.944; *p* = 0.203]. Moreover, despite a statistically significant main effect of complexity level [*X*^2^(2) = 15.296; *p* < 0.001], when compared two by two, the levels did not appear significantly different, even for levels 1 and 3 [*T*_(740)_ = 1.809; *p* = 0.071]. This could be explained by the small effect size (*W* = 0.055).

To summarize, WP5 was an important dimension, since whatever the CT, MWL from WP5 appeared the highest compared to the other WP dimensions. This result was coherent with the logic of the tasks which were carried out on a screen with visual processing of the information. Moreover, our protocol with three complexity levels generated three distinct levels of WP5 for NBT and Corsi. This was the case for both WP3 for Corsi and WP4 for WCST. WP7 was the only dimension that was not influenced by the complexity level whatever the CT.

Moreover, for each WP dimension, there were significant differences between tasks. Concerning WP3, DT and Corsi were significantly greater than GNG. This result was logical since these two tasks solicited visuo-spatial memory. With regard to WP4, NBT and WCST generated the highest MWL compared to the other CTs. It was logical because these tasks implicitly led the participant to say mentally or out loud the information to keep in mind. Finally, WP7 was the only CT without a main effect of the task, showing consistency since all participants had similar manual output (interactions limited to keystrokes or mouse/touchpad movements).

### Clustering subjective MWL and verifying the correlation between subjective classes and complexity level

For these analyses, we used *JASP* software (version JASP 0.16).

We used an unsupervised classification method, the K-means technique with the Hartigan-Wong algorithm, a default parameter in *JASP* software. We observed the quality control of the clusters with three criteria. The first two were the Akaike Information Criterion (AIC) and the Bayesian Information Criterion (BIC) which were analytical methods for estimating the relevancy of statistical models to each other for a given data set (the lower the values the better the clustering results). Also, we added the Silhouette score that compared the mean intra-cluster distance to the mean nearest-cluster distance (ranging from −1 to 1, where 1 represented a perfect score). Obtaining several quality criteria was critical since they could nuance the results.

#### Variables selection for K-means clustering

The selection of subjective dimensions for each task is summarized in Table 1.

**Table 1:** Selection of dimensions of the WP used by K-means clustering for each task.

	**WP3-spatial**	**WP4-verbal**	**WP5-visual**	**WP7-manual**
N-back task (NBT)		**X**	**X**	
Corsi	**X**		**X**	**X**
Go/No-Go (GNG)			**X**	**X**
WCST	**X**	**X**	**X**	
Dual task (DT)	**X**	**X**	**X**	

First, WP5 was selected for all tasks due to its dimension importance. Whatever the CT, MWL from WP5 turned out the highest compared to other WP dimensions (Cf. *WP questionnaire in the Results section*).

Concerning NBT, the retained WP dimensions were WP4 and WP5. Indeed, Conover's *post hoc* comparisons indicated that MWL based on WP3 for NBT turned out significantly lower than other CTs for all the tasks (except GNG). Moreover, NBT presented a set of letters in the center of the screen without spatial movement of the stimuli, and the manual interactions were limited to the directional pad (left and right arrow only) and were not part of the logic of the task (only verbal working memory).

With regard to Corsi, the retained WP dimensions were WP3, WP5, and WP7. The Corsi task showed a lower WP4 score than all the other tasks. Furthermore, the verbal dimension was not supposed to come into play, given that the task was based on visual-spatial memory.

For GNG, the retained WP dimensions were WP5 and WP7. We discarded WP3 as it presented a set of stimuli in the center of the screen (black disks and black squares) without spatial movement of these stimuli. We also discarded WP4, given that GNG appeared significantly lower than NBT and WCST (tasks involving verbal processing).

Concerning WCST, the retained WP dimensions were WP3, WP4, and WP5. We discarded WP7 given that manual interactions were not part of the logic of the task (which implied mostly mental flexibility).

With regard to DT, the retained WP dimensions were WP3, WP4, and WP5. The DT solicited visual-spatial memory (WP3) during the Corsi test and verbal dimension during the calculation task. We discarded WP7 given that all its levels did not turn out significantly different (Cf. Supplementary Table 4).

Once the WP dimensions selection step was over, we realized a K-means clustering of the data with all the selected subjective variables (Cf. Table 1) to determine the number of proposed clusters associated with the quality criteria scores. We, therefore, performed K-means clustering in three ways. The first aimed to perform the K-means model, optimizing it by the Silhouette score followed by BIC and AIC values with a limit of 10 clusters (default setting on *JASP* software). Then, we compared these scores with another way of clustering data: fixing K = 3 clusters.

Accordingly, for each selected subjective variable, we obtained 4 × 3 scores with 4 ways of clustering and 3 clustering quality criteria (Cf. Supplementary Table 5).

First, for the analysis of the results, for each quality—for instance, the AIC value - we identified among the selected subjective variables (Cf. Table 1) the one that gave the best AIC value score (gray cells in Supplementary Figure 5). Then, in a second step, we identified the selected subjective variables, fixed at K = 3, which gave the two best AIC values. Green cells and light blue cells were respectively the best selected subjective variables (based on AIC) fixed at 3, and the second best. This method allowed for the selection of the selected subjective variables showing the best scores for K = 3 compared to the scores obtained in the same conditions but with an optimized K.

Then, we wanted to determine whether the MWL values classified in each of the three classes of clustering were obtained in the conditions believed to induce these MWL classes. Thus, we examined if a high correlation occurred (ranging from “0”- no correlation to “100”- best correlation) between the assignment of MWL data in an MWL class by K-means and the data assignment according to complexity levels.

Concerning NBT, we compared the reliability of rankings based on different combinations of selected subjective variables from complete NASA-TLX, WP4, and WP5. For each variable selection, the clustering quality criteria for each clustering technique had been considered beforehand, once optimized by the Silhouette score followed by the BIC and AIC values. Comparing the AIC values (the lower the values the better the clustering results) when the classification was fixed at three clusters or optimized by AIC, the results ranged from 513.520 (shaded cell in Supplementary Table 5) to 790.680. Moreover, among the fixed K = 3, the NASA/WP5 selection obtained the lowest AIC score (thus the best score) with 644.160 (which was relatively close to 513.520, the best score with AIC optimization). Concerning BIC value (the lower the values the better the clustering results) with the K = 3 fixed, the NASA/WP5 selection obtained the lowest BIC score with 708.600 (which was relatively close to 667.610, the best score with BIC optimization). Concerning the Silhouette score (ranging from −1 poor clustering to 1 very good clustering) with the fixed K = 3, the selection NASA/WP4 obtained the highest Silhouette score (thus the best score) with 0.230 (which was relatively close to 0.290, the best score with Silhouette optimization). Therefore, we were able to obtain three classes of MWL classes with high-quality indicators for the NASA/WP4 and NASA /WP5 selections.

Concerning the correlation coefficient (Table 2) between MWL classes with K-means and MWL classes with complexity levels, we obtained respectively 0.64 and 0.65 for NASA/WP4 and NASA/WP5 selections.

**Table 2:** Correlation coefficient (Spearman) between MWL classes through K-means and complexity levels.

	**NBT**						**GNG**					
**Subjective combination**	**NASA** + **WP5**			**NASA** + **WP4**			**NASA** + **WP5**			**NASA** + **WP7**		
Complexity level	1	2	3	1	2	3	1	2	3	1	2	3
MWL0	37	16	1	25	8	0	20	4	0	20	3	0
MWL1	15	32	26	27	37	20	31	33	13	31	34	14
MWL2	1	5	26	1	8	33	2	16	40	2	16	39
Correlation coefficient	**0.65**			**0.64**			**0.65**			**0.65**		
	**Corsi**										
Subjective combination	**NASA** **+** **WP3**			**NASA** **+** **WP5**			**NASA** **+** **WP3** **+** **WP5**				
Complexity level	1	2	3	1	2	3	1	2	3		
MWL0	41	6	0	46	6	0	43	5	0		
MWL1	12	43	20	7	44	22	10	45	22		
MWL2	0	4	33	0	3	31	0	3	31		
Correlation coefficient	**0.79**			**0.82**			**0.8**				
	**WCST**						**DT**					
Subjective combination	**NASA** **+** **WP3**			**NASA** **+** **WP5**			**NASA** **+** **WP3**			**NASA** **+** **WP5**		
Complexity level	1	2	3	1	2	3	1	2	3	1	2	3
MWL0	34	6	4	34	6	4	39	2	0	39	1	0
MWL1	17	41	37	17	42	37	13	36	17	14	37	18
MWL2	2	6	12	2	5	12	1	15	36	0	15	35
Correlation coefficient	**0.5**			**0.51**			**0.75**			**0.76**		

Concerning Corsi, we compared different selected subjective variables based on complete NASA-TLX, WP3, WP5, and WP7. With regard to the AIC value with K = 3 fixed (Supplementary Table 5), the selection NASA/WP3 obtained the lowest AIC score with 573.400 (which was relatively close to 457.160, the best score with AIC optimization). For the BIC value with K = 3 fixed, the selection NASA/WP3 obtained the lowest BIC score with 637.840 (which was relatively close to 609.490, the best score with BIC optimization). In contrast, the Silhouette score with fixed K = 3, the selection NASA/WP3/WP5 obtained the highest Silhouette score with 0.260 (which was relatively close to 0.340, the best score with Silhouette optimization). We could also notice that NASA/WP5 selection consistently ranked second while being very close to the results of NASA/WP3. Therefore, three classes of MWL classes could be obtained with high-quality indicators for NASA/WP3, NASA /WP5, and NASA/WP3/WP5 selections. Concerning the correlation coefficient (Table 2) between MWL classes with K-means and MWL classes with complexity levels, we obtained respectively 0.79, 0.82, and 0.80 for NASA/WP3, NASA/WP5, and NASA/WP3/WP5 selections.

**Figure 9:**
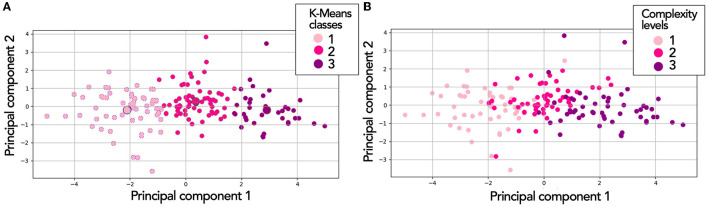
Three MWL classes (based on K-means) in Principal Component Analysis (PCA) space for the Corsi test. In **(A)**, visualization is independent of complexity level. In **(B)**, the same distribution of points [as in **(A)**], but through complexity level.

As an example, Figure 9 shows visually the overlap between the three clusters of subjective MWL (with NASA-TLX/WP5) for the Corsi test. The results were presented in a PCA (Principal Component Analysis) space, as the representation in the PCA space allowed to reduce the number of dimensions of the data (there were 7 dimensions of MWL).

Thus, Figure 9A shows the three MWL classes based on K-means. Figure 9B shows the same distribution of points (as Part A) but through complexity level. We could notice that between the two techniques, there was a good agreement in the positioning of statistical individuals in the MWL classes. However, there was little or even no overlapping of points in Part A. Concerning Part B, we observed some overlaps, particularly at the boundaries adjacent to level 2.

Concerning GNG, we compared different selected subjective variables based on complete NASA-TLX, WP5, and WP7. In the AIC value and BIC value with K = 3 fixed (Supplementary Table 5), the selection NASA/WP5 obtained the lowest AIC and BIC scores. In the Silhouette score with the fixed K = 3, the selection NASA/WP7 obtained the highest Silhouette score. Regarding the correlation coefficient (Table 2) between MWL classes with K-means and MWL classes with complexity levels, we obtained 0.65 for both NASA/WP5 and NASA/WP7 selections.

With regard to WCST, we compared different selected subjective variables based on complete NASA-TLX, WP3, WP4, and WP5. For all clustering quality criteria, NASA/WP5 selection showed the best scores with K = 3 fixed, closely followed by NASA/WP3 selection (Supplementary Table 5). Moreover, when clustering was obtained by the Silhouette score, K-means proposed K = 3 for these two-variable selections. Therefore, three classes of MWL classes with high-quality indicators could be obtained for NASA/WP3 and NASA/WP5. For the correlation coefficient (Table 2) between MWL classes with K-means and MWL classes with complexity levels, we obtained respectively 0.50 and 0.51 for NASA/WP3 and NASA/WP5 selections.

Finally, concerning DT, we compared different selected subjective variables based on complete NASA-TLX, WP3, WP4, and WP5. For all clustering quality criteria, NASA/WP5 selection showed the best scores with K = 3 fixed, closely followed by NASA/WP3 selection (Supplementary Table 5). Therefore, without complexity levels, three MWL classes with high-quality indicators could be obtained for NASA/WP3 and NASA/WP5 selections. For the correlation coefficient (Table 2) between MWL classes with K-means and MWL classes with complexity levels, we obtained respectively 0.75 and 0.76 for NASA/WP3 and NASA/WP5 selections.

In conclusion, we could obtain three classes of subjective MWL for each of the five CTs with high-quality criteria, without considering complexity levels. When each quality criterion between tasks was compared, the scores varied relatively little. Regarding the AIC value, Corsi showed the best score (573.400) and WCST the worst (705.550). For the BIC value, Corsi showed the best score (637.840) and WCST the worst (769.990). Concerning the Silhouette score, GNG showed the best score (0.270), closely followed by Corsi (0.260), and DT was the worst (0.180). Moreover, WCST was the only task that proposed natural K = 3 clusters with Silhouette score optimization for several selected subjective variables. Hence, for all three clustering quality criteria, Corsi appeared systematically in the lead of the CTs and WCST often appeared in the bottom.

Furthermore, WCST (for all selected subjective variables) was the CT with the lowest correlation between MWL classes with K-means and MWL classes with complexity levels (≤ 0.51), unlike Corsi and DT which showed the highest correlation (>0.70).

### MWL classification based on complexity levels and performance or performance only

#### Procedure

For these analyses, we used the programming language Python (version Python 3.9.7). We only presented the best result between the selected subjective variables from previous statistics to avoid overloading the reading.

**Figure 10:**
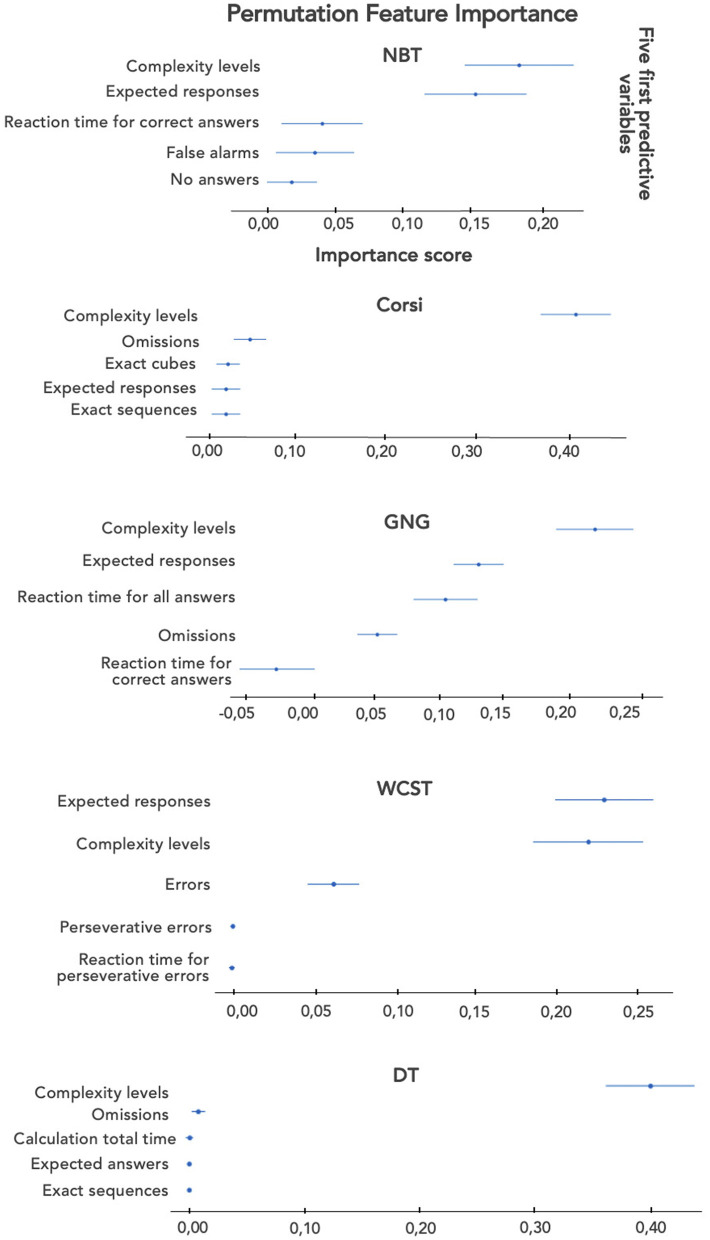
Bar graphs on the importance of the five first performance variables for each task.

With regard to NBT, NASA-TLX/WP4, with balanced accuracy, our model could determine subjective MWL based on *Complexity level, False alarms, Omissions*, and *Reaction time for all items* with the best cross-validation score equal to 57.6% (±3.64%). Then, the importance of the different variables through the PFI (all performance variables combined) was considered, and the accuracy score for NASA-TLX/WP4 was calculated (Figure 10). The LDA model was mainly based on *Complexity level* and *Expected responses* for the prediction. Without *Complexity level*, the accuracy of the model was equal to 36.12% (±2.64%) with five NBT performances: *No answers, Expected responses, False alarms, Omissions*, and *Reaction time for all items*.

Concerning Corsi, NASA-TLX/WP5, with balanced accuracy, our model could determine subjective MWL based just on *Complexity level* with the best cross-validation score equal to 79.97% (±0.0%). In Figure 10, the LDA model for NASA-TLX/WP5 is mainly based on *Complexity level* for the prediction. Without *Complexity level*, the accuracy of the model was equal to 53.28% (±3.48%) with four Corsi performances: *False cubes, Omissions, Exact sequences*, and *Total Time*.

Concerning GNG, NASA-TLX/WP5, with balanced accuracy, our model could determine subjective MWL based on *Complexity level, Expected responses*, and *Omissions* with the best cross-validation score equal to 51.35% (±1.75%). The LDA model for NASA-TLX/WP5 was based on *Complexity level, Expected responses*, and *Reaction time for all answers* for the prediction (Figure 10). Without *Complexity level*, the accuracy of the model was equal to 47.74% (±1.9%) with three GNG performances: *Reaction time for correct answers, Reaction time for all answers*, and *Expected responses*.

With regard to WCST, NASA-TLX/WP5, with balanced accuracy, our model could determine subjective MWL based on *Complexity level* and the *Number of errors* with the best cross-validation score equal to 53.86% (±1.64%). The LDA model for NASA-TLX/WP5 was based on *Complexity level* and *Expected responses* for the prediction (Figure 10). Without *Complexity level*, the accuracy of the model was equal to 42.88% (±3.43%) with four WCST performances: *Number of errors, Perseverative errors, Reaction time for all items*, and *Expected responses*.

Finally, for DT, NASA-TLX/WP5, with balanced accuracy, our model could determine subjective MWL based on *Complexity level* with the best cross-validation score equal to 73.71% (±0.0%). The LDA model for NASA-TLX/WP5 was mainly based on *Complexity level* for the prediction (Figure 10). Without *Complexity level*, the accuracy of the model was equal to 44.74% (±5.66%) with six DT performances such as *False cubes, Omissions*, or *Expected responses*.

In conclusion, we were able to predict subjective MWL far beyond the chance threshold (equal to 33%) when considering *Complexity level* and performance as variables for all tasks. It seems relevant to note that the best prediction results were based on the selected subjective variables NASA-TLX/WP5 for all CTs except NBT. Therefore, this dimension turned out to be the most relevant one to consider for establishing the classification model. Moreover, Corsi and DT were the tasks with a cross-validation score higher than 73% (±0.0%). With *Complexity level*, the MWL prediction model relied predominantly on *Complexity level* alone (according to the PFIs). The other tasks performed less well, but the importance of the predictive variables was not focused mostly on the *Complexity level*.

Thus, when considering models only based on performance measures to predict MWL, the validation score decreased for Corsi and DT, but they remained above the chance threshold. Moreover, the validation scores also decreased for the other tasks, and Corsi remained the best candidate with a cross-validation score appearing higher than 50%. NBT was the worst with an accuracy lesser than 40%.

## Discussion

This study aimed to test several candidate cognitive tasks (CTs) with distinct complexity levels. We then relied on combined statistical methods to answer the two following questions:

1- Can we identify subjective mental workload classes corresponding to the complexity levels?
2- Can we predict these mental workload classes based on complexity levels and/or performance?

### Conception of cognitive tasks with three complexity levels

We aimed to obtain three complexity levels in terms of impact on performance (*Expected responses*). As expected, NBT proved to be in line with previous studies (Arvaneh et al., 2015; Dimitrakopoulos et al., 2017; Ries et al., 2018). In this context, NBT served initially as a benchmark to calibrate the complexity levels of the other tasks. As expected, for at least two tasks (Corsi and WCST), several complexity levels generated several *Expected responses* levels. Concerning GNG and DT, the last complexity levels were not different in terms of performance. These results could be explained by the levels of requested solicitations which were too close between the last levels for the two tasks. For example, in the DT, adding a multiplication (highest complexity level) to a subtraction (intermediate level) did not impact performance. This result was not in line with So et al. (2017) study that obtained significant differences in performance measures between an intermediate level of subtraction and a higher level of multiplication. However, the So et al. (2017) study showed a limit as it mixed multiplication and division operations during the last level, making the impact of the two operations difficult to distinguish. Perhaps a division would have given more significant differences. Nevertheless, based on our pre-test calibration phase, we could observe that division would have led to a too high difficulty concerning the other tasks.

In conclusion and to reach our first objective, the priority was to obtain three complexity levels that generated three levels of *Expected responses*, which NBT, Corsi, and WCST demonstrated. Next, we aimed to know if these complexity levels also generated three MWL classes.

### Tasks complexity level to induce MWL classes

Concerning the definition of MWL, which is a multidimensional concept, we systematically considered two questionnaires from two models of MWL: NASA-TLX and WP questionnaires.

With regard to WP dimensions, for some tasks, all complexity levels generated distinct classes of MWL, like WP3-*Spatial processing* for the Corsi.

However, no WP dimension could discriminate all MWL classes for GNG and DT. These results contradicted the Rubio study (Rubio et al., 2004) which observed that WP was the only questionnaire to reveal differences due to the task complexity. Nevertheless, despite the least discrimination to complexity level, the WP questionnaire appeared worthwhile when measuring the proportion of the attentional resource used in a particular sensory-motor dimension as specified in Tsang and Velazquez (1996). For example, in our study, Corsi and DT were the CTs generating the highest MWL based on spatial processing. These results are in with the logic of the tasks based on spatial memory.

Concerning NASA-TLX, all complexity levels generated distinct levels of overall MWL for all CTs. However, across tasks, non-equivalence occurred between the overall MWL classes. Thus, the levels obtained were relative, not absolute, and therefore depended on the task despite quite close “orders” of values. Moreover, for the overall MWL classes of all tasks, the averages varied between approximately 100 and 300 on a scale of 600. Thus, the three levels of overall MWL were not in the intermediate MWL zone but in the first half of the MWL, within a low and intermediate zone of MWL. Even though we didn't expect this result, it made us consider that the tasks offered allowed us to reach levels of MWL being fairly close but nevertheless distinguishable and often difficult to obtain with subjective scales. Once this level of accuracy in discriminating MWL classes is potentially reached, we could add a complexity level to the tasks. For this purpose, the Corsi test was the best candidate since, among all the tasks, it allowed the most distinct overall NASA-TLX levels. This was the first argument for not considering the NBT as a reference task for MWL measurement and modeling.

Regarding the measures of each of NASA-TLX dimensions, they demonstrated that the dimensions contributed differently to the overall MWL score. This result was in line with previous findings (Rubio et al., 2004; Fallahi et al., 2016; Longo, 2017) where the overall score did not allow the detection of subtle variations. Nevertheless, NASA-TLX dimensions could be used to determine which dimensions were pulling up or down the overall NASA-TLX. For example, for all complexity levels of our NBT, *Physical demand* turned out the lowest score compared to the other NASA-TLX dimensions. This result was in line with the study by Malakoutikhah et al. (2021) which also compared the six dimensions of NASA-TLX for NBT and observed that *Physical demand* had the lowest scores compared to the other NASA-TLX dimensions. It was coherent with the solicitations of our task since manual interactions were limited to keystrokes or mouse/touchpad movements.

This study also confirmed the complementarity of the questionnaires based on different methodological or theoretical approaches. While the WP questionnaire concerned the saturation of the multiple pools of attentional resources, the NASA-TLX questionnaire concerned, in part, the impact of external factors on subjective MWL.

Concerning our hypothesis **H1**, we could conclude that our protocol provided different overall MWL classes concerning the complexity level. To this aim, Corsi and DT were the best tasks and WCST was the worst. Indeed, the NASA-TLX questionnaire is known to show a high correlation with performance (Rubio et al., 2004). This may explain why MWL based on NASA-TLX did not allow three distinct levels for all dimensions of GNG for which performances were not impacted by the third complexity level. However, through WCST which had three distinct levels of performance but not three levels of overall MWL, we could observe the limit of using only performance for MWL measurement. Performance could sometimes degrade as the complexity level increased, but MWL did not increase linearly. In our study, WCST turned out to be a perfect example of the non-linear relationship between subjective MWL and performance.

### Correspondence between MWL classes considering the complexity level

To ensure that the three complexity levels of each task could allow distinct classes of MWL, we decided to consider the correlation between MWL classes of values obtained with a clustering method (considering only subjective measures of MWL as variables) and MWL classes of values obtained with complexity levels.

First, among the CTs, Corsi allowed the best clustering, that is, the most distinct MWL classes, this task being the one for which the most dimensions of MWL allowed this distinction between classes. Hence the interest in considering all the dimensions of MWL rather than just the overall value as some authors did (Radüntz, 2017; Guan et al., 2021).

Moreover, if we compared the MWL classes obtained *via* the labels of complexity level or the clustering, we obtained a good correspondence, more particularly for Corsi. Thus, our **H1** was confirmed. And the Corsi test allowed the best grouping into MWL classes. Moreover, these classes corresponded well to what was expected *via* the complexity levels. These results were the second argument for not considering the NBT as a reference task. Furthermore, as far as we knew, no previous study had ascertained the observation of three distinct classes of MWL by combining two analytics methods as we did by considering the correspondence between MWL classes with K-means and MWL classes with complexity levels. Our approach of considering the correlation between two different ways of grouping data was a first in the scientific literature.

### Proposal of a predictive model of MWL classes

The Corsi test turned out to be the best task to predict MWL with complexity levels and performance. Therefore, this task was well-dimensioned in terms of complexity levels. Thus, Corsi demonstrated a high level of reliability to induce a priori (at least at the beginning of the activity) the class of MWL in which the person should be. This result was the third argument for not considering the NBT as a reference task. Using NBT as a reference to establish an MWL model, as several authors (Dimitrakopoulos et al., 2017; Beh and Wu, 2021; Malakoutikhah et al., 2021) did, could explain the increasing number of studies using it to obtain several levels of MWL. A selectivity bias due to the number of NBT-based studies for MWL could have been introduced. With our analysis, we pointed out the limits of NBT. Concerning the Corsi test, we found different MWL classes, and these classes were correlated to those obtained by considering the MWL data *via* the complexity level. Moreover, for Corsi, we could propose a model based on performance and complexity levels predicting MWL with good accuracy. Furthermore, Corsi had simple instructions and was not socially marked, as people who cannot read and write or people with different alphabets could use it. Finally, it appeared to be a CT that allowed constant overall MWL gaps and provided a high number of possibilities to easily modify the complexity levels. As a result, among our five CTs, the Corsi test proved to be the best candidate to fulfill our first objective.

### A model to predict mental workload only with performance

For the second objective of this study, we aimed to obtain a “real-time” indicator to identify the “shifts” of MWL during the activity. This was another critical aspect of our work as we knew that MWL evolved during the activity due to its multidimensional nature. Thus, we decided to focus only on performance data during the task, neglecting physiological ones considered difficult to interpret and with validity problems. Therefore, we tried to circumvent the limits of the correspondence between MWL and performance data by favoring the number and thus the sensitivity of the performance measurements performed. Nevertheless, when the MWL predictive models were only based on performance measures without including the complexity level, the results decreased for all the tasks despite the result remaining quite good for Corsi and DT. It seems relevant to note that Corsi was the only task with an accuracy appearing higher than 50% (which was above the chance threshold of about 33%). The number of performance dimensions can explain this result. Corsi and DT had the best predictions and had performance dimensions higher than the other tasks (7 and 9 for Corsi and DT respectively). Thus, multiplying the performance data was a good way to make this real-time indicator more reliable and sensitive. Thus, **H2** was confirmed.

Nevertheless, even if the prediction level was still much higher than the chance level for these two tasks, adjusting the complexity level only on these models did not seem sufficient. These results were in line with studies indicating that performance measures could not, of themselves, describe MWL, since the operators could potentially vary efforts to maintain a constant performance level (Reid and Nygren, 1988; Raufaste et al., 2004; Cain, 2007; Radüntz, 2017). Although performance measures could account for MWL during the activity, we must be aware of their main limit. Thus, to improve our model, we should carry it out on other measures of MWL. Therefore, even though those psychophysiological measures had several constraints, they could be worthwhile to improve our models. It could indeed become possible to triangulate the three categories of measures (subjective, performance, and psychophysiological) to better understand a person's MWL (Charles and Nixon, 2019; Longo et al., 2022). As a matter of fact, considering MWL through the prism of these three categories of measures would overcome the limitations of each measure. Future studies measuring MWL through physiological measures should require a reference model of subjective MWL being close to the essence of MWL (Hart and Staveland, 1988) like our model which was based on two complementary models of subjective MWL.

### Contribution to the body of knowledge

Firstly, it is widely accepted that MWL is a *multidimensional concept* (Hancock et al., 2021), and our results support this by showing that the dimensions vary depending on the activity. Indeed, the NASA-TLX and WP questionnaires are complementary since they are sensitive to different sources of MWL. Thus, we were able to demonstrate that the cognitive tasks in our study induced different MWL classes (with differences according to the task) depending on the complexity level of the activity (mental demands, time pressure), the strategies used (performance, mental effort) and the individual's emotional state (frustration). However, some dimensions measured by the WP did not appear to show significant variation across tasks, suggesting that cognitive tasks more or less saturate the attentional resources mobilized in each resource pool of Wickens' model (Wickens, 1984, 1987, 2008). Therefore, we confirm that MWL can be defined as *the degree of activation of a finite pool of resources, limited in capacity, while cognitively processing a primary task over time*. Thus, the variations in the dimensions of MWL solicited as a function of the tasks accrediting that MWL enables individuals to *cope with static task demands, by devoted effort and attention* (Longo et al., 2022). Moreover, our results confirmed that for the same task demand, the profile of solicitation and dimensions of MWL are task-specific. Therefore, it is essential to carefully choose the type of measure used based on the task's characteristics to avoid missing any effects by selecting an unsuitable questionnaire.

Secondly, we have demonstrated that performances can serve as a predictor of MWL, supporting models that establish a link between task demands, MWL, and performance (Hart and Staveland, 1988; De Waard, 1996; Young et al., 2015). Nevertheless, we nuanced these models so that performance predictors must be sufficiently numerous and varied to be correctly linked to changes in MWL. Our results also support models that emphasize the contribution of exogenous factors like the *stress/strain* model (Karasek, 1979; Raufaste et al., 2004). Thus, the prediction of MWL increased for all the tasks when we included the complexity level (corresponding to task demands) as a predictor.

Thirdly, we proposed a protocol that can induce a desired MWL class at the beginning of an activity, at least for the Corsi test. Our results suggest that the NBT may not be a suitable reference task to model the MWL, as is currently done in some studies (Dimitrakopoulos et al., 2017; Ries et al., 2018; Beh and Wu, 2021; Malakoutikhah et al., 2021). Among our five CTs, Corsi proved to be a better candidate as it reliably induced different MWL classes, allowing us to predict the expected MWL.

## Conclusion

We tested five context-free tasks, all providing three distinct levels of overall MWL. Given the advantages of combined statistical methods, we could evaluate the distinction of three MWL classes associated with three complexity levels. Among our five CTs, the Corsi test obtained different MWL classes reliably enough to make predictions about the expected MWL. Consequently, this study provides a foundation for future research aimed at predicting the MWL class before the activity and adjusting the complexity level to keep the desired MWL class. For this purpose, multiplying the performance data was a good way to obtain a more reliable and sensitive real-time indicator. This recommendation could also be applied to the learning context with the aim to measure or predict cognitive load. Thus, our future objective is to improve this model with psychophysiological measurements (like EEG data) of MWL available in real-time during a task.

### Limitations

Our experiment was conducted in a remote online environment. The results could be different when tasks are performed in person. Moreover, the subjective MWL model could be enriched with other factors that could influence MWL such as internal factors (emotions or expertise) or external factors (the design). Besides, the impact of the design on MWL is a research question currently being studied within our team.

## Data availability statement

The raw data supporting the conclusions of this article will be made available by the authors, without undue reservation.

## Ethics statement

The studies involving human participants were reviewed and approved by the CERNI (in French) for the Ethical Committee for Non-Interventional Research of the University of Nantes. The patients/participants provided their written informed consent to participate in this study.

## Author contributions

L-EL wrote the protocol with inputs from IM-P. SR was responsible for the technical and computational work of the study. L-EL conducted the experiment. Signal processing, data analysis, and interpretation were performed by L-EL, IM-P, SM, and TL. The manuscript was written by L-EL with inputs from IM-P. All authors read and approved the final manuscript.
