# Supplementary material for: Cognitive tasks and combined statistical methods to evaluate, model, and predict mental workload
Source: Front Psychol. 2023 May 12;14:1122793. doi: 10.3389/fpsyg.2023.1122793 (PMC10213687; doi:10.3389/fpsyg.2023.1122793)
Supplement: Supplementary file 1 [file Data_Sheet_1.DOCX]

Supplementary Material

**Cognitive tasks and combined statistical methods to evaluate, model, and predict mental workload**

**L.E., Louis^1-2^, S., Moussaoui^2^, A., Van Langhenhove^3^, S. Ravoux^1^, T., Le Jan^1-2^, V., Roualdes^3^, I., Milleville-Pennel^2^**

*1 Entreprise onepoint, 3 rue Lavoisier, 44100 Nantes, France*

*2 Nantes Université, École Centrale Nantes, CNRS, LS2N, UMR 6004, Nantes, F-44000, France*

*3 Department of Neurosurgery, CHU (Centre Hospitalier et Universitaire) Nord Laënnec, Nantes Saint-Herblain, France*

***** Correspondence:** Lina-Estelle “Linelle” Louis

[linaestelle.louis@gmail.com](mailto:linaestelle.louis@gmail.com)

**Expected responses**

**A**

**
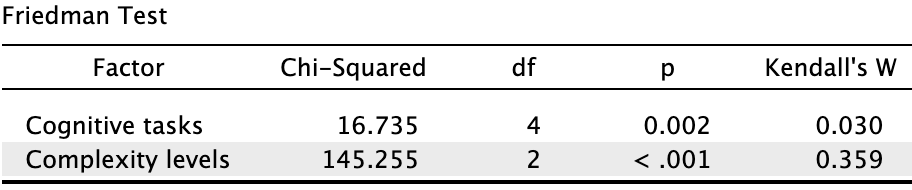
**

**B**

**
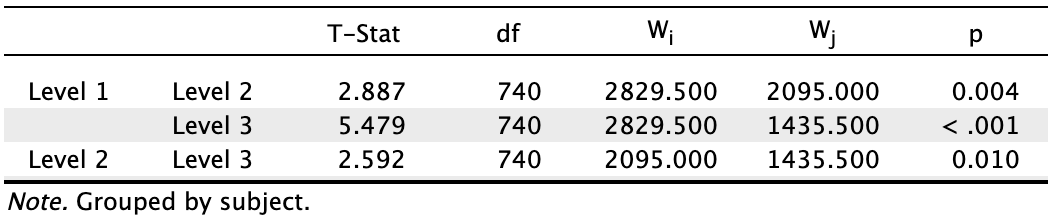
**

**Cv**


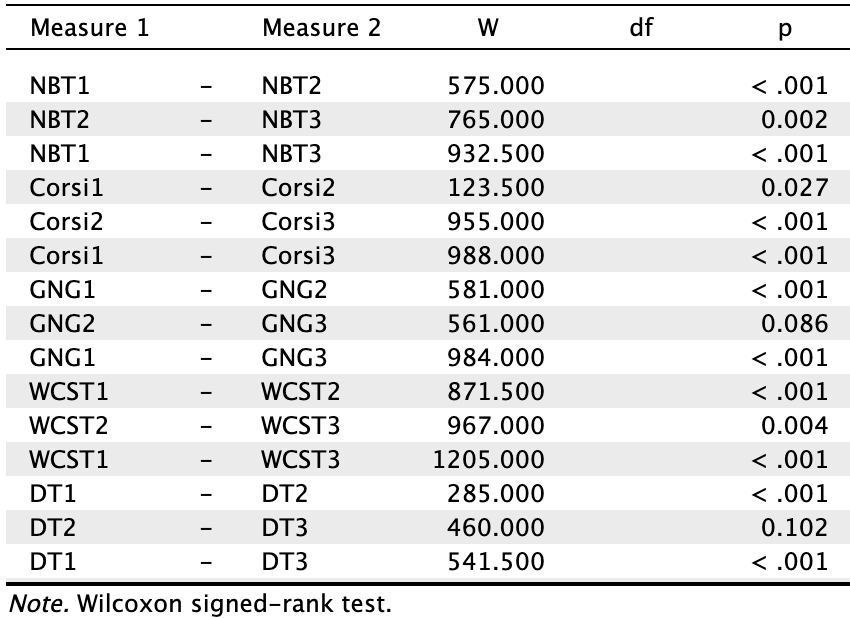


**Supplementary Table 1:** Performance measures in terms of *Expected responses*. A) Non-parametric Repeated-Measures ANOVA with Friedman test for the two main effects, cognitive tasks, and complexity levels. B) Independently of the task, Conover’s post hoc comparisons for the three complexity levels. C) Task by task, post‐hoc paired sample Wilcoxon signed-rank tests of complexity levels.

**Overall NASA-TLX**

**A**

**
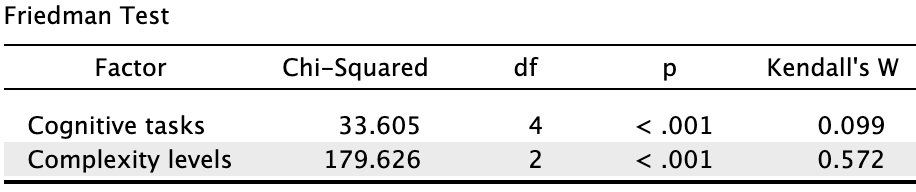
**

**B**

**
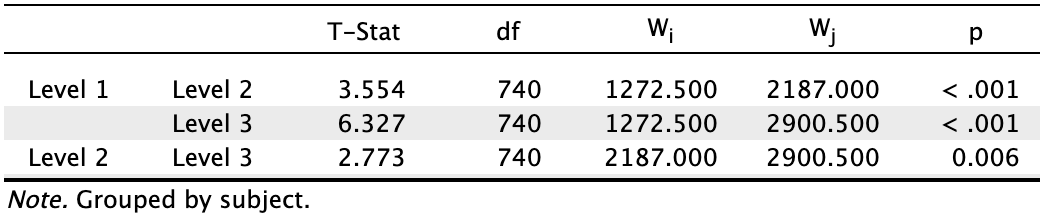
**

**Cv**

**
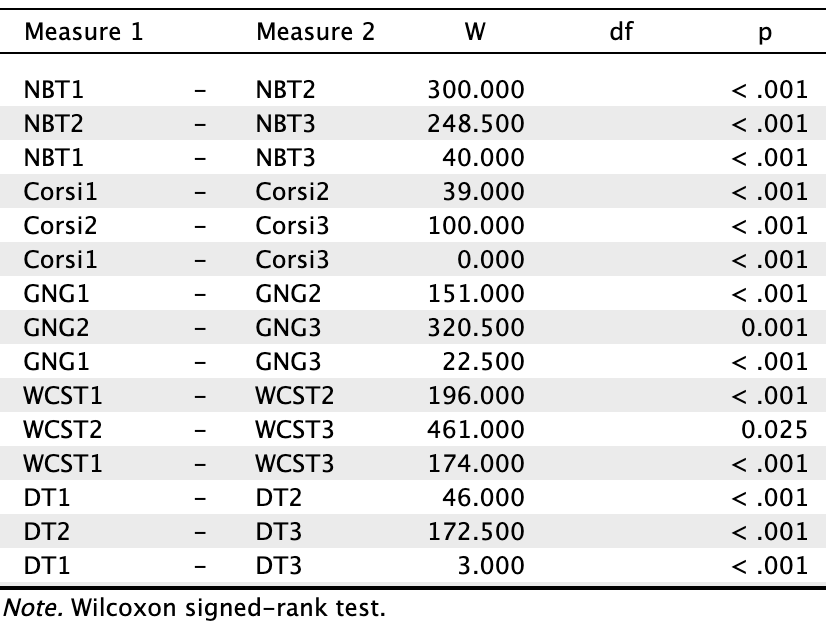
**

**
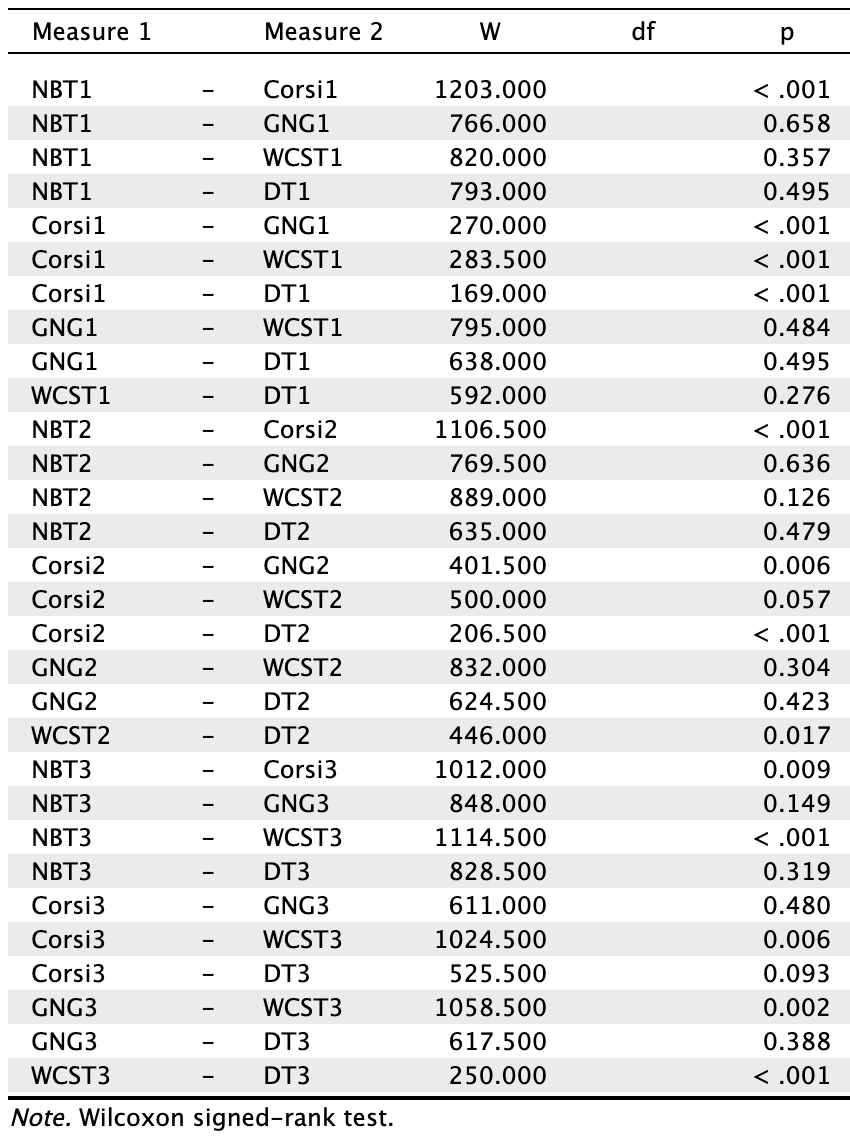
**

**Dv**

**Supplementary Table 2:** Overall NASA-TLX. A) Non-parametric Repeated-Measures ANOVA with Friedman test for the two main effects, cognitive tasks, and complexity levels. B) Independently of the task, Conover’s post hoc comparisons for the three complexity levels. C) Task by task, post‐hoc paired sample Wilcoxon signed-rank tests of complexity levels. D) Level by level, post‐hoc paired sample Wilcoxon signed-rank tests of cognitive tasks.

**Mental demand**


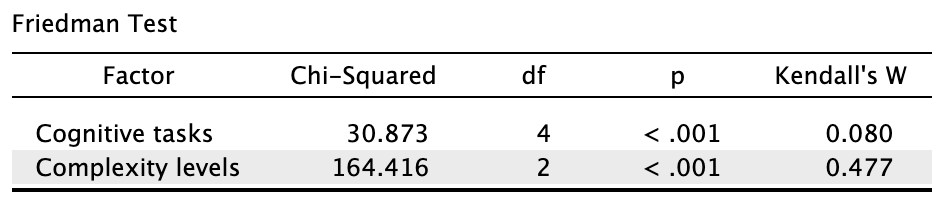


**A**

**B**


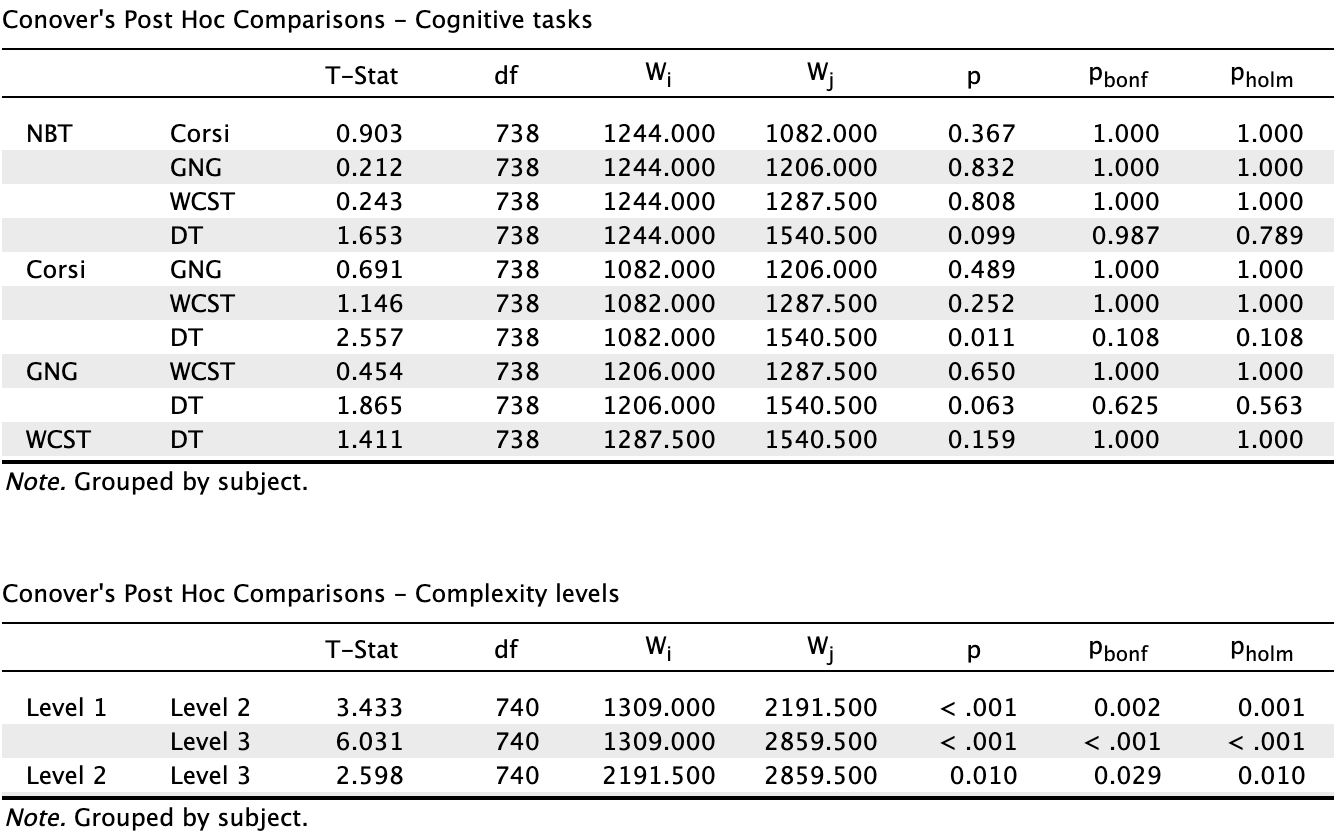


**C**


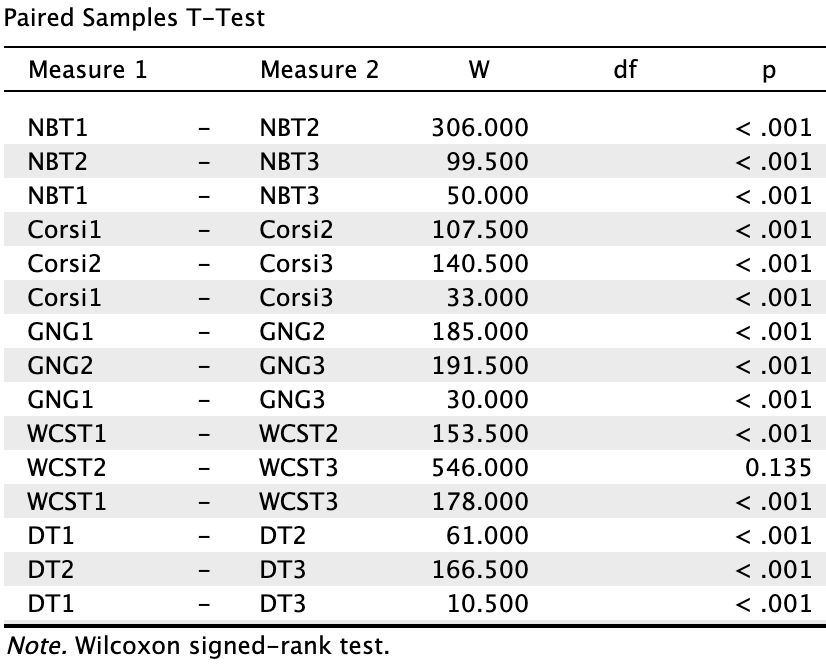


**Physical demand**

**A**


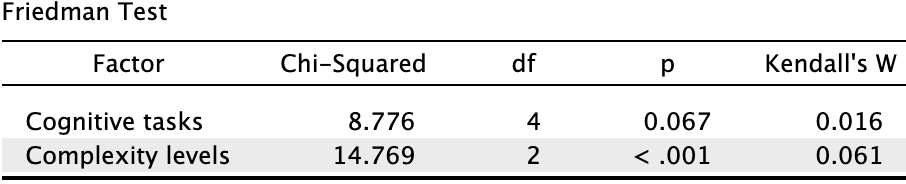


**B**


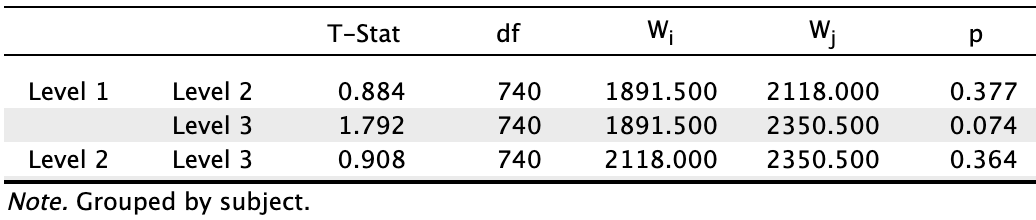


**C**


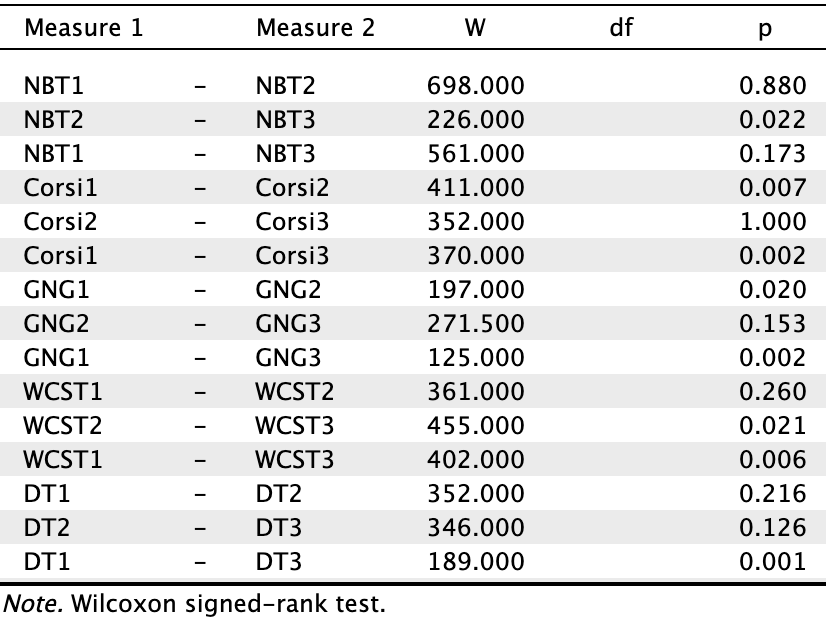


**Temporal demand**

**A**


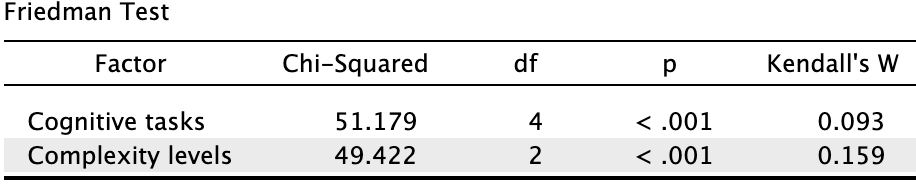


**B**


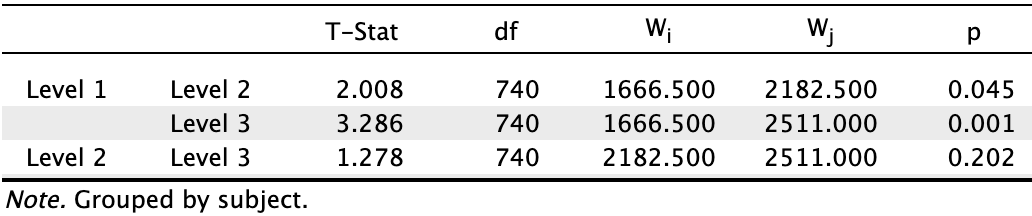


**C**


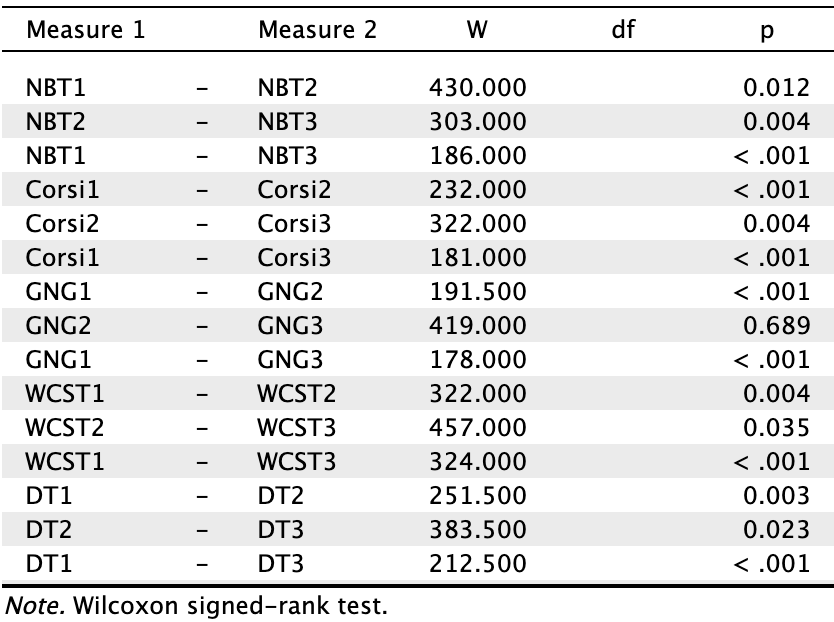


**Effort**

**B**

**A**


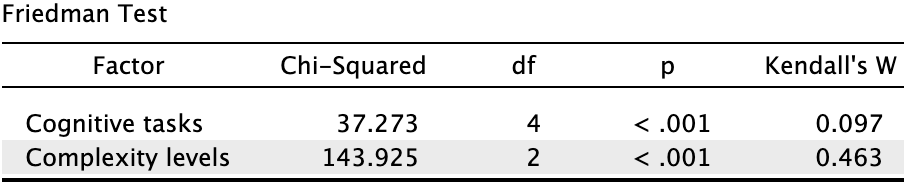


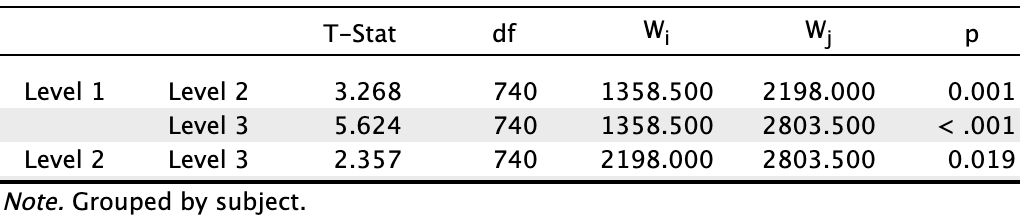


**C**


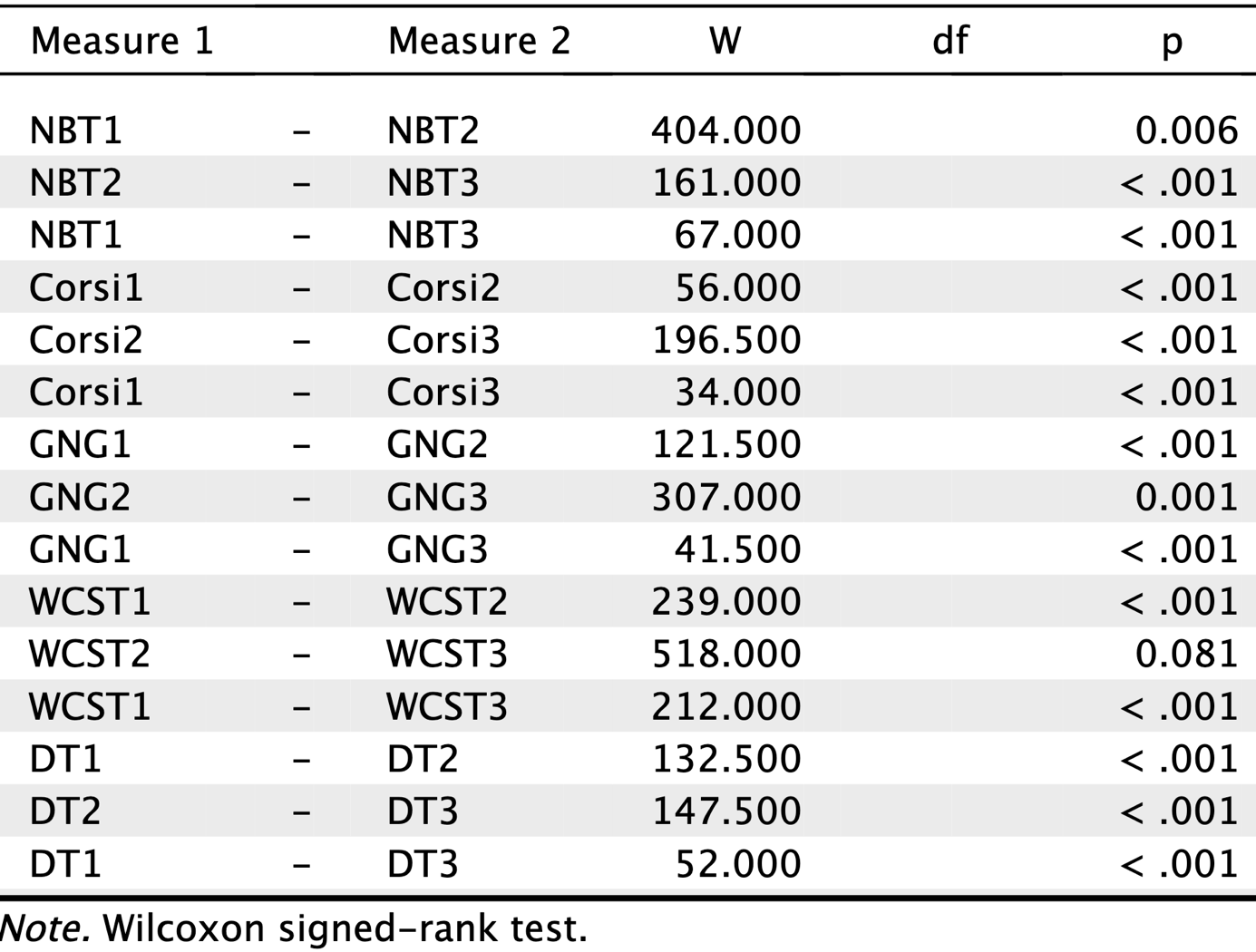


**Performance**

**A**

**
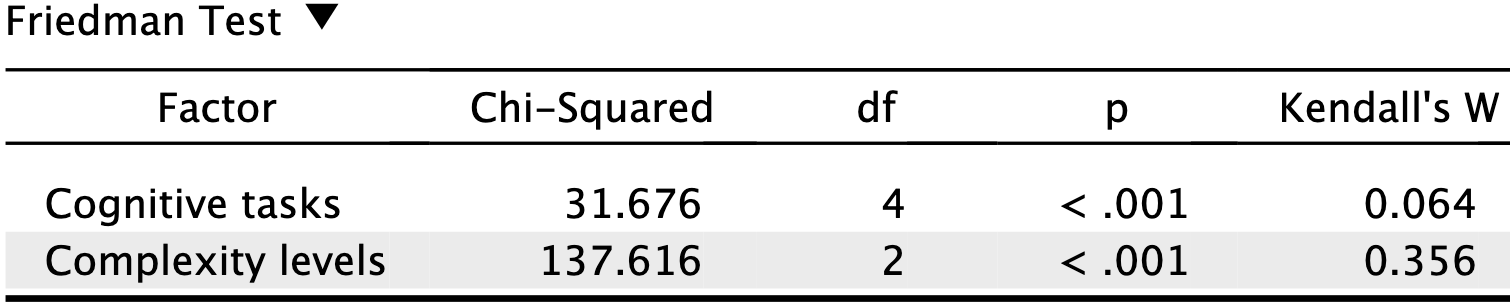
**

**B**

**
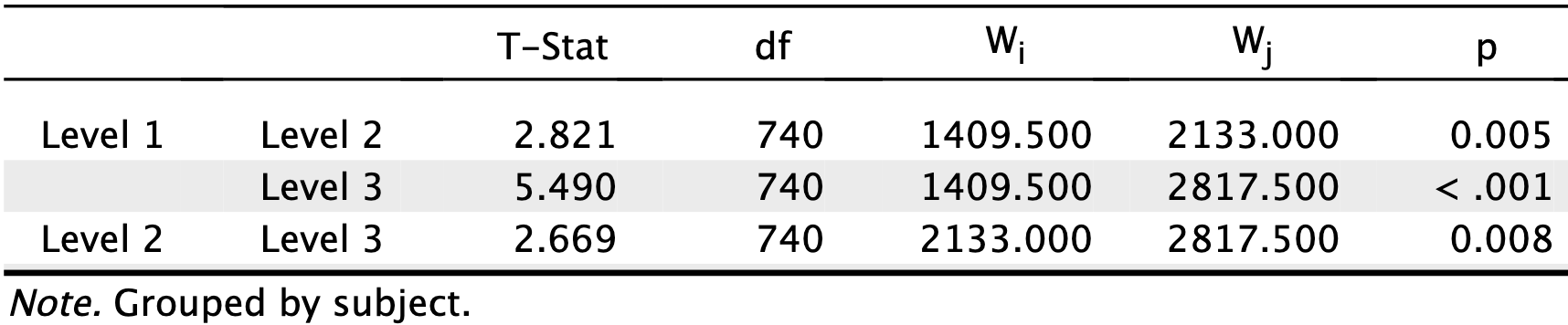
**

**C**

**
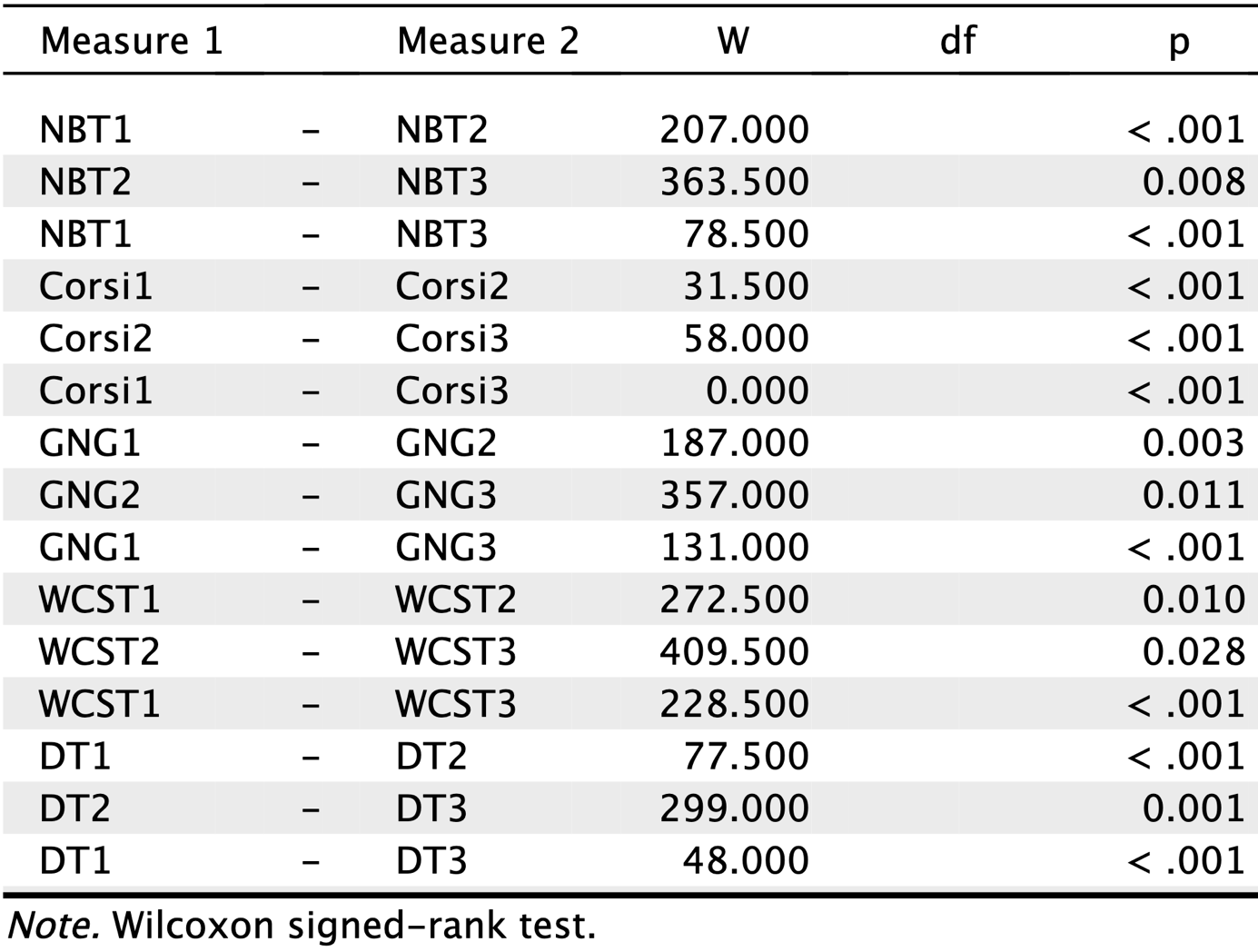
**

**Frustration**

**A**


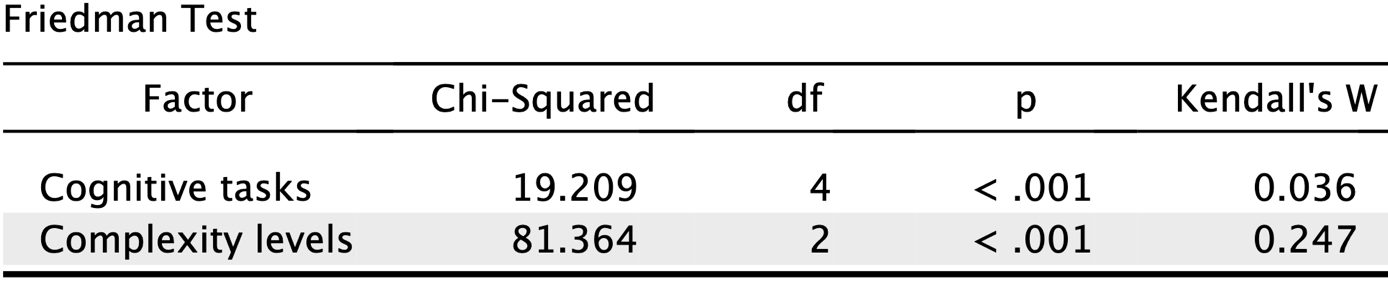


**B**


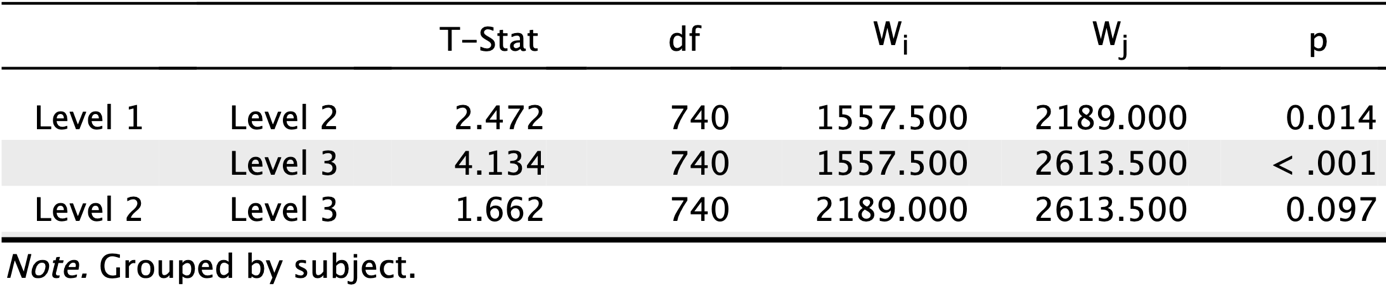


**C**


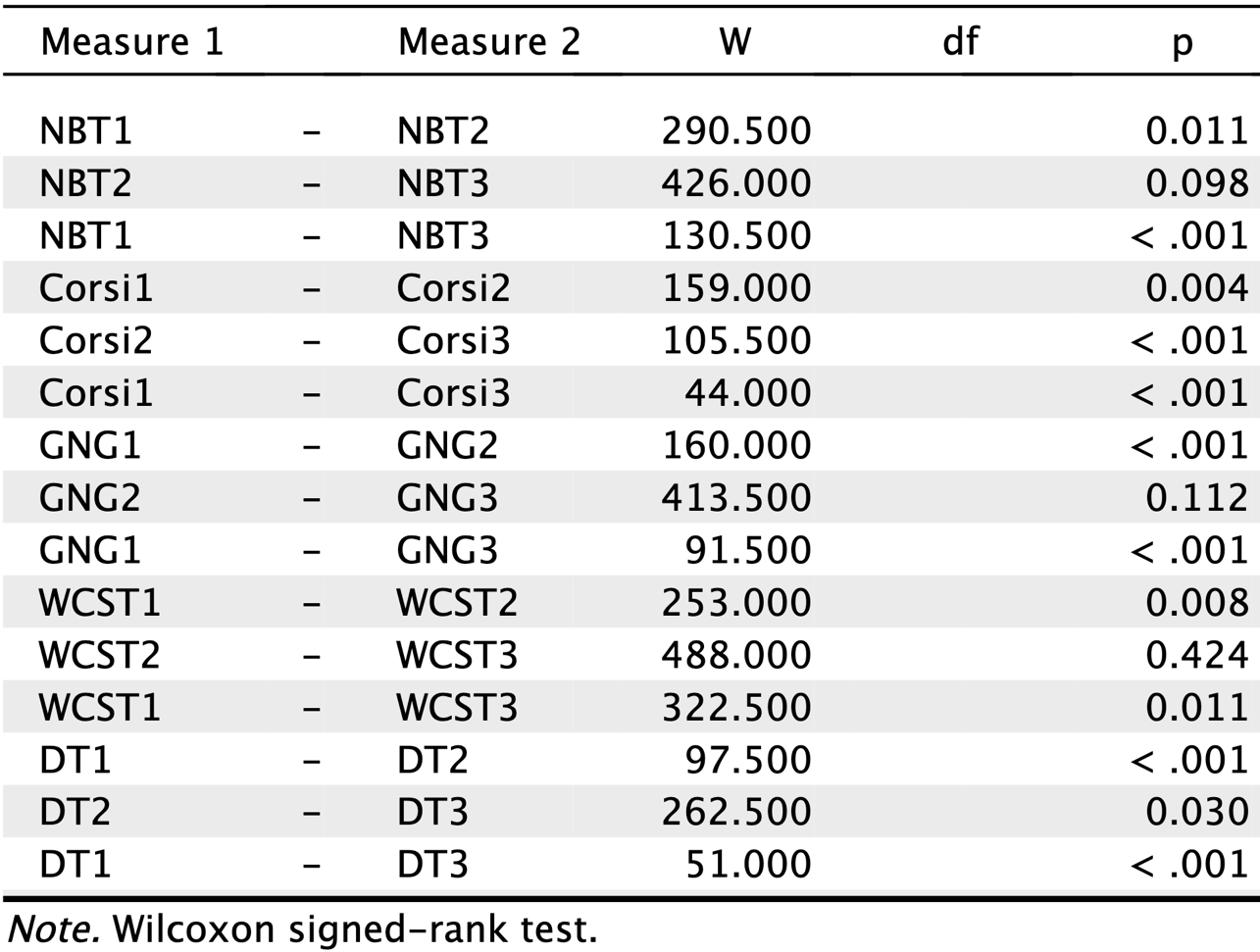


**Supplementary Table 3:** Dimensions of NASA-TLX questionnaire. A) Non-parametric Repeated-Measures ANOVA with Friedman test for the two main effects, cognitive tasks, and complexity levels. B) Independently of the task, Conover’s post hoc comparisons for the three complexity levels. C) Task by task, post‐hoc paired sample Wilcoxon signed-rank tests of complexity levels.

**WP3**

**A**

**
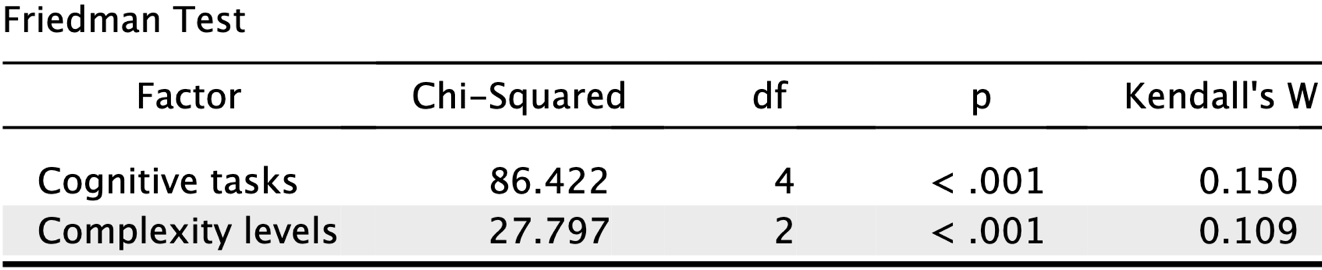
**

**B**

**
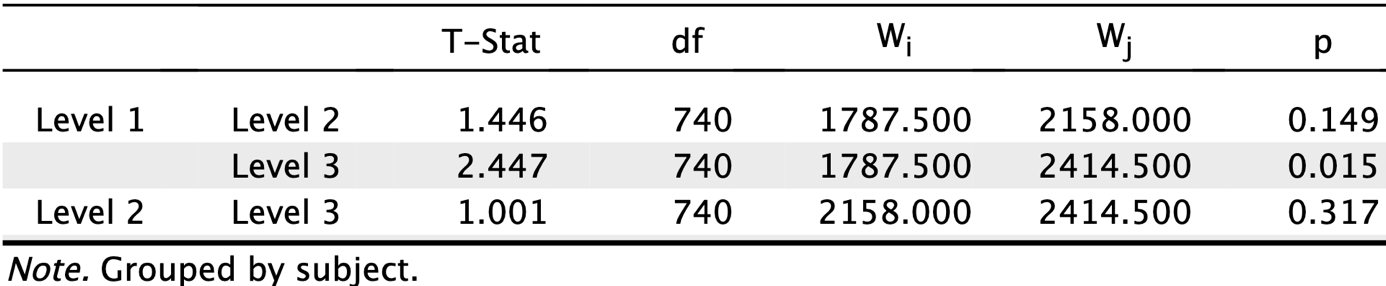
**

**C**

**
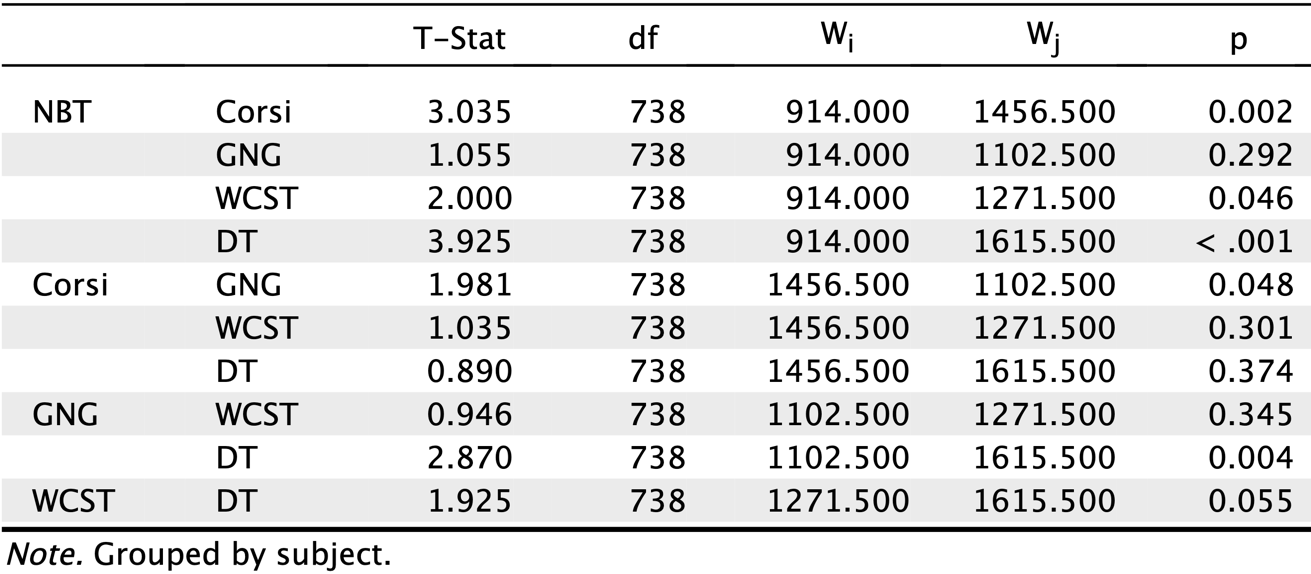
**

**D**

**
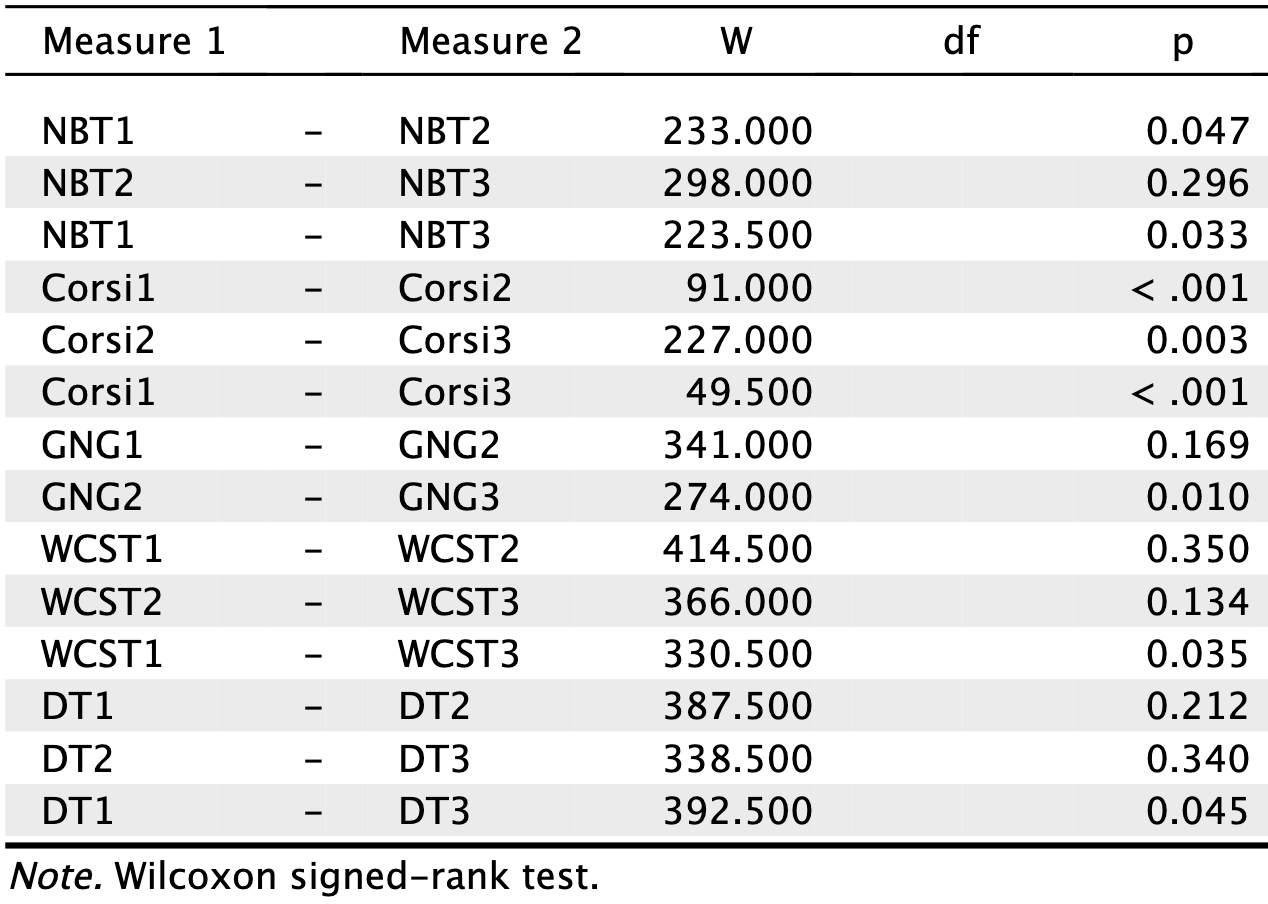
**

**WP4**

**C**

**A**

**D**


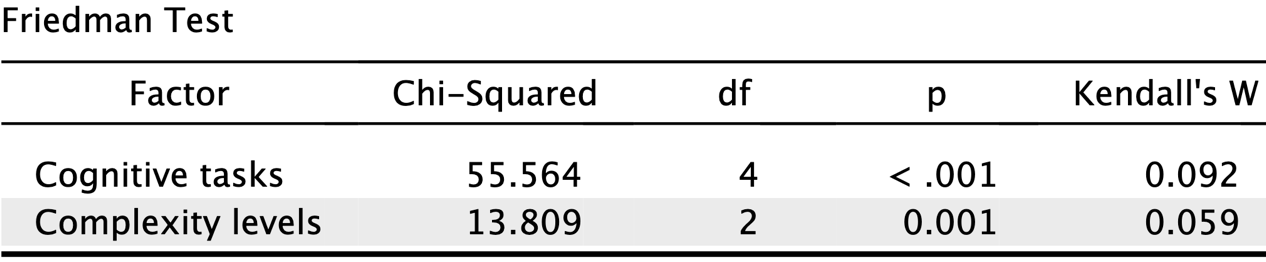


**B**


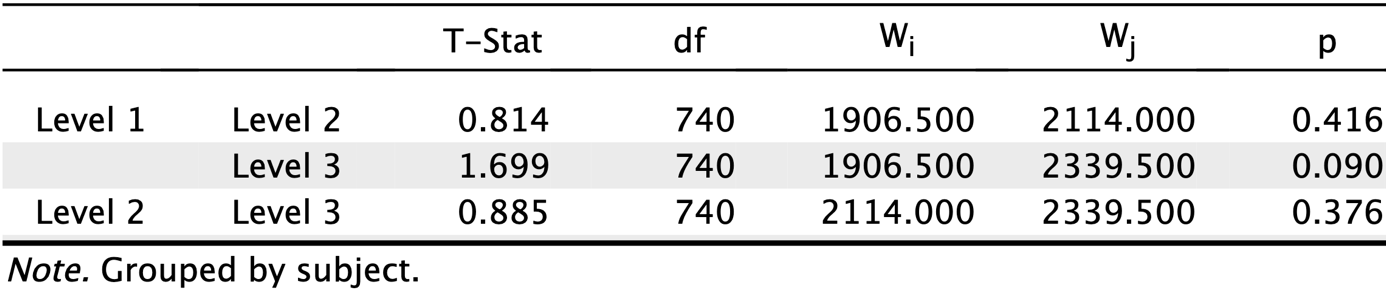


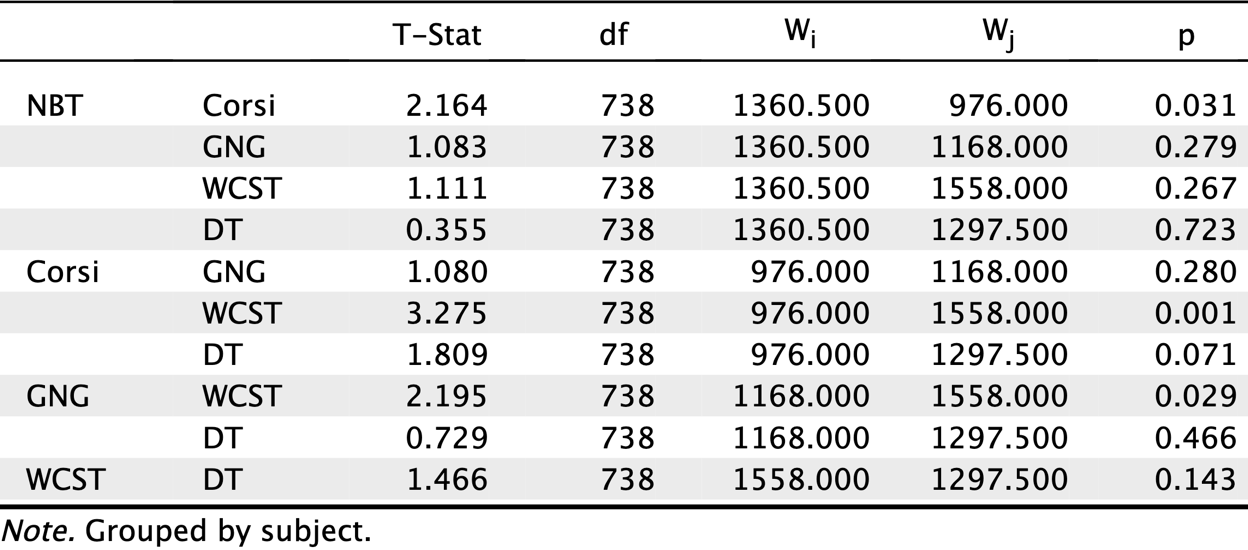


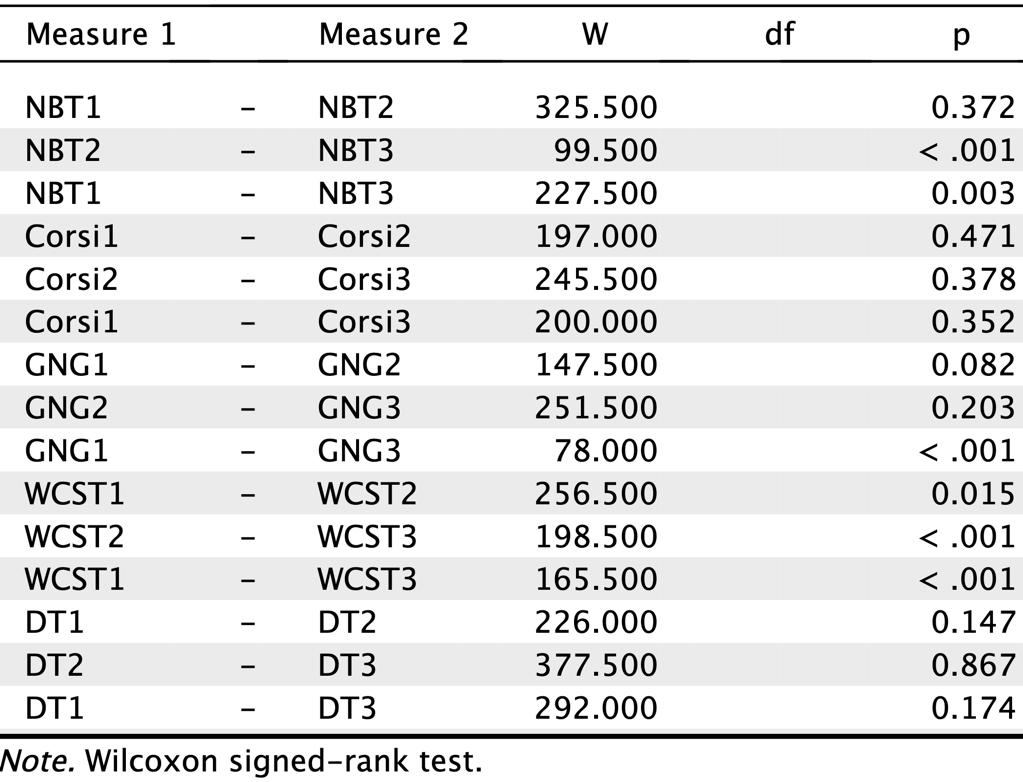


**WP5**

**A**

**
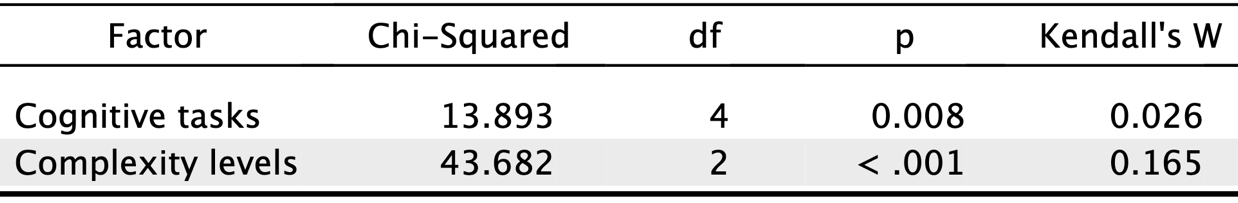
**

**B**

**
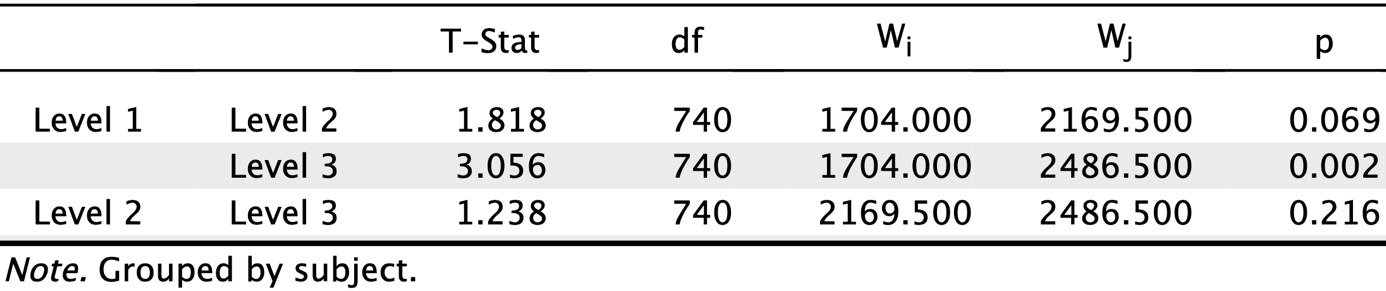
**

**C**

**
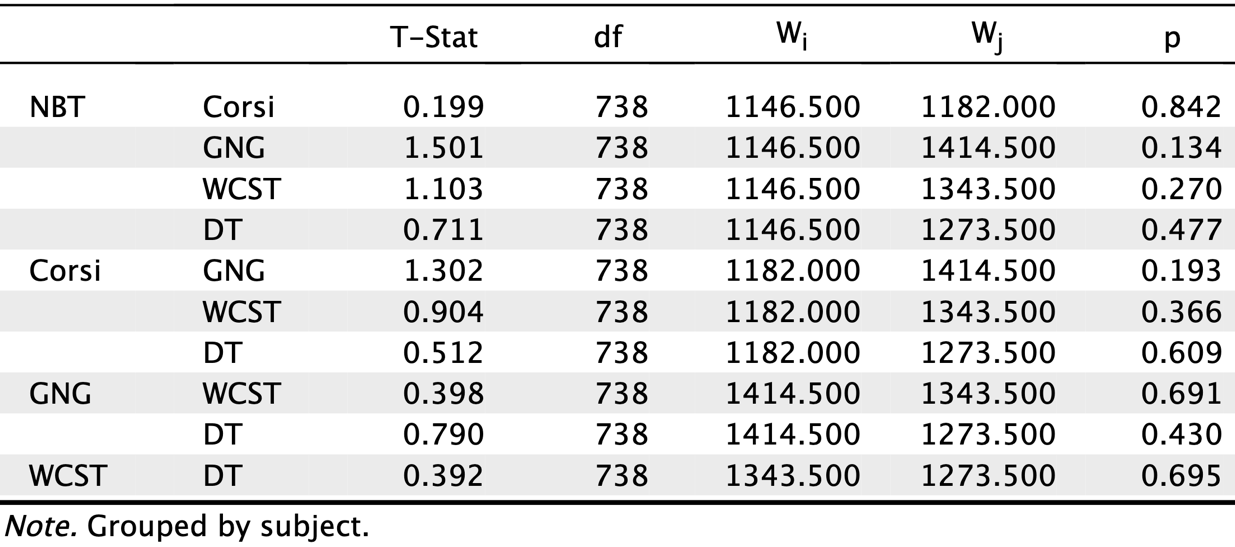
**

**D**

**
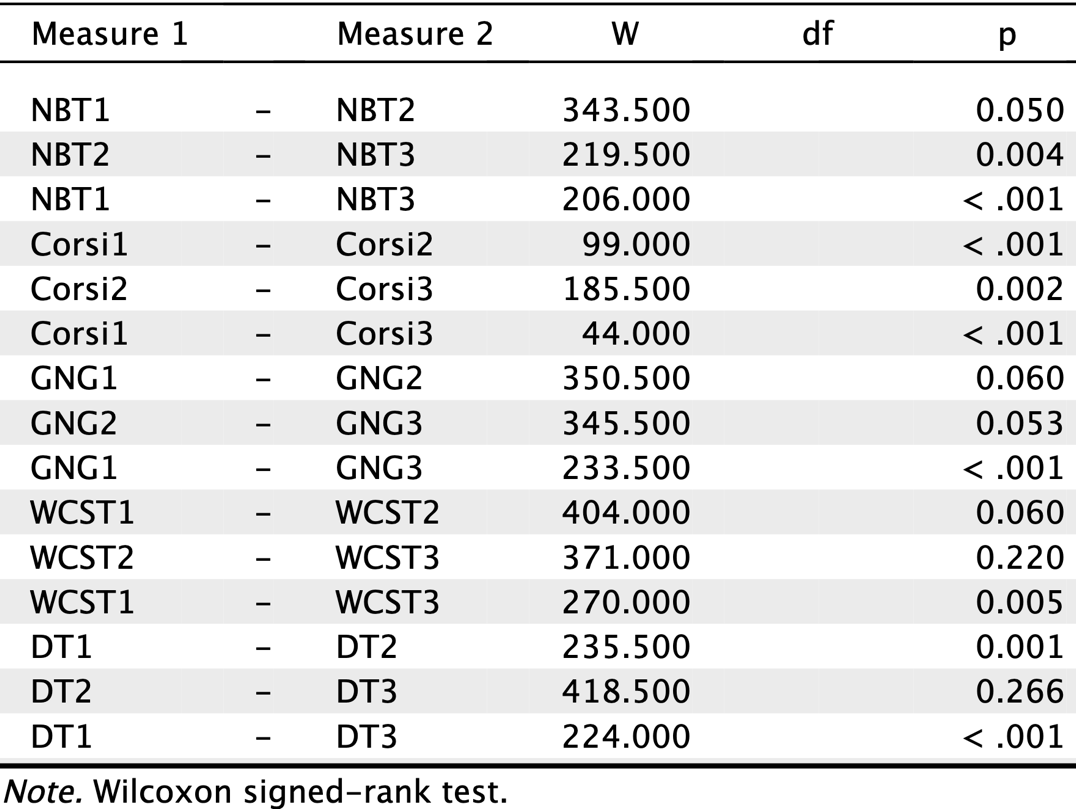
**

**WP7**

**A**

**
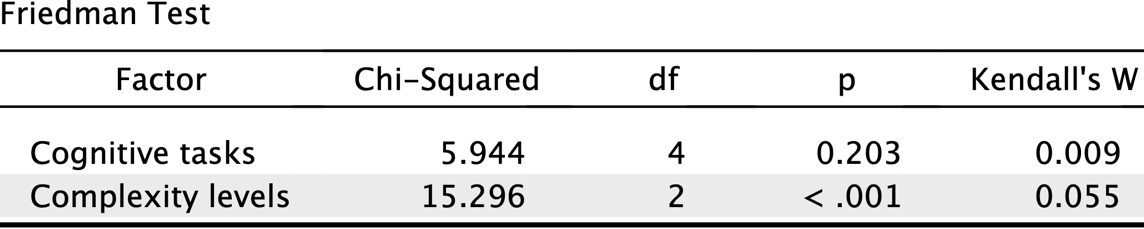
**

**B**

**
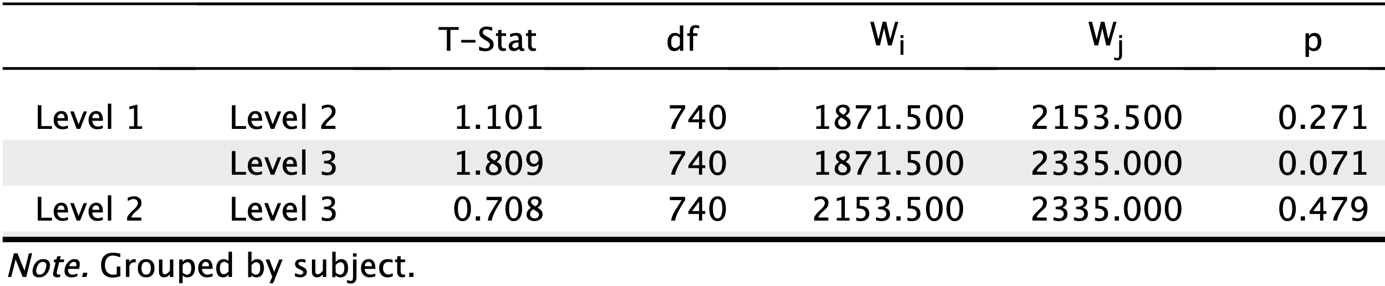
**

**D**

**
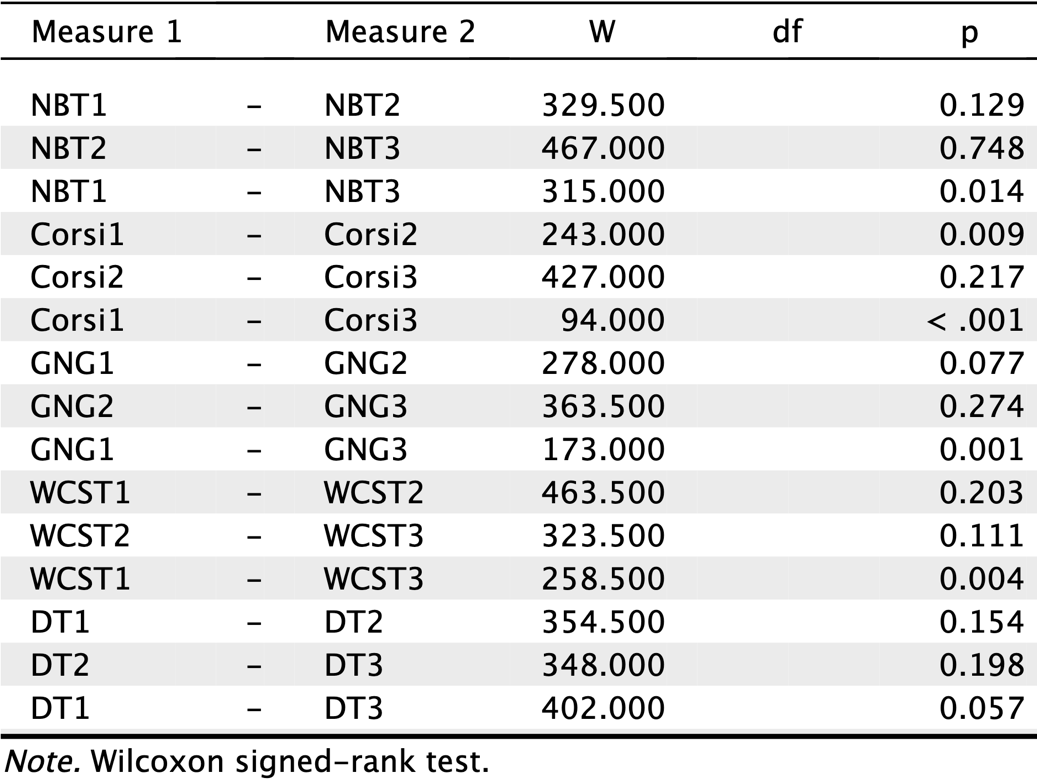
**

**Supplementary Table 4:** Dimensions of WP questionnaire. A) Non-parametric Repeated-Measures ANOVA with Friedman test for the two main effects, cognitive tasks, and complexity levels. B) Independently of the task, Conover’s post hoc comparisons for the three complexity levels. C) Independently of the complexity level, Conover’s post hoc comparisons for the five cognitive tasks. D) Task by task, post‐hoc paired sample Wilcoxon signed-rank tests of complexity levels.


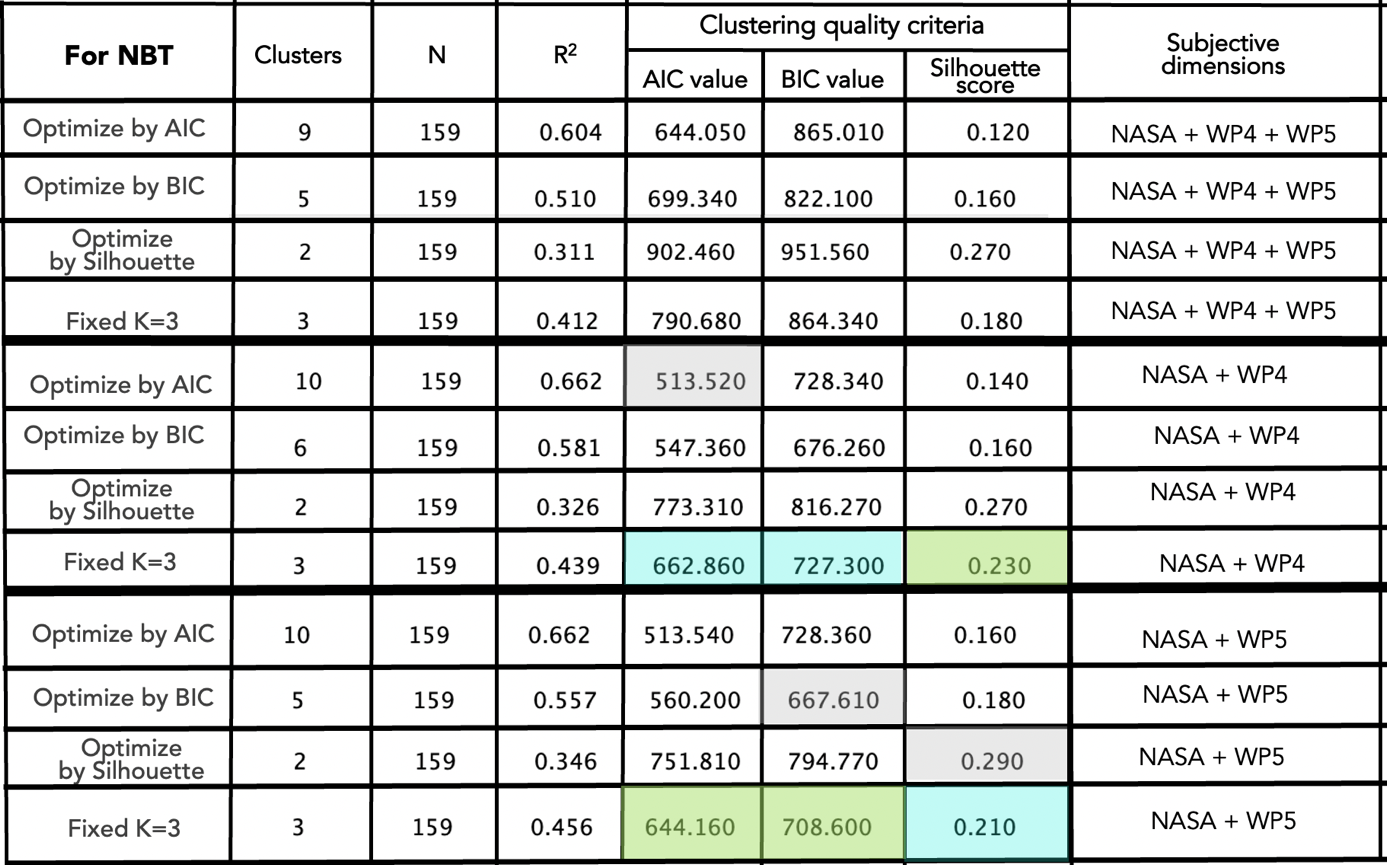


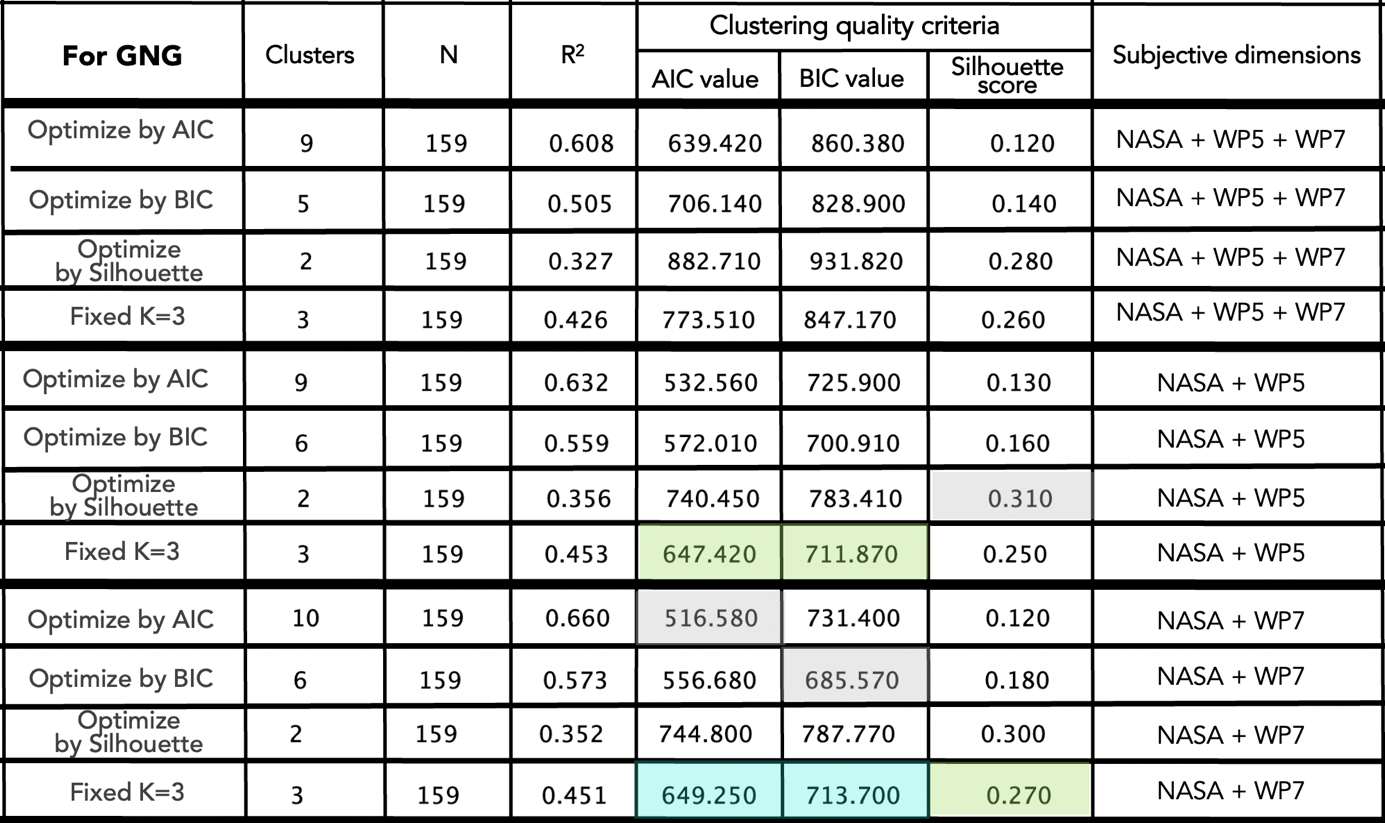


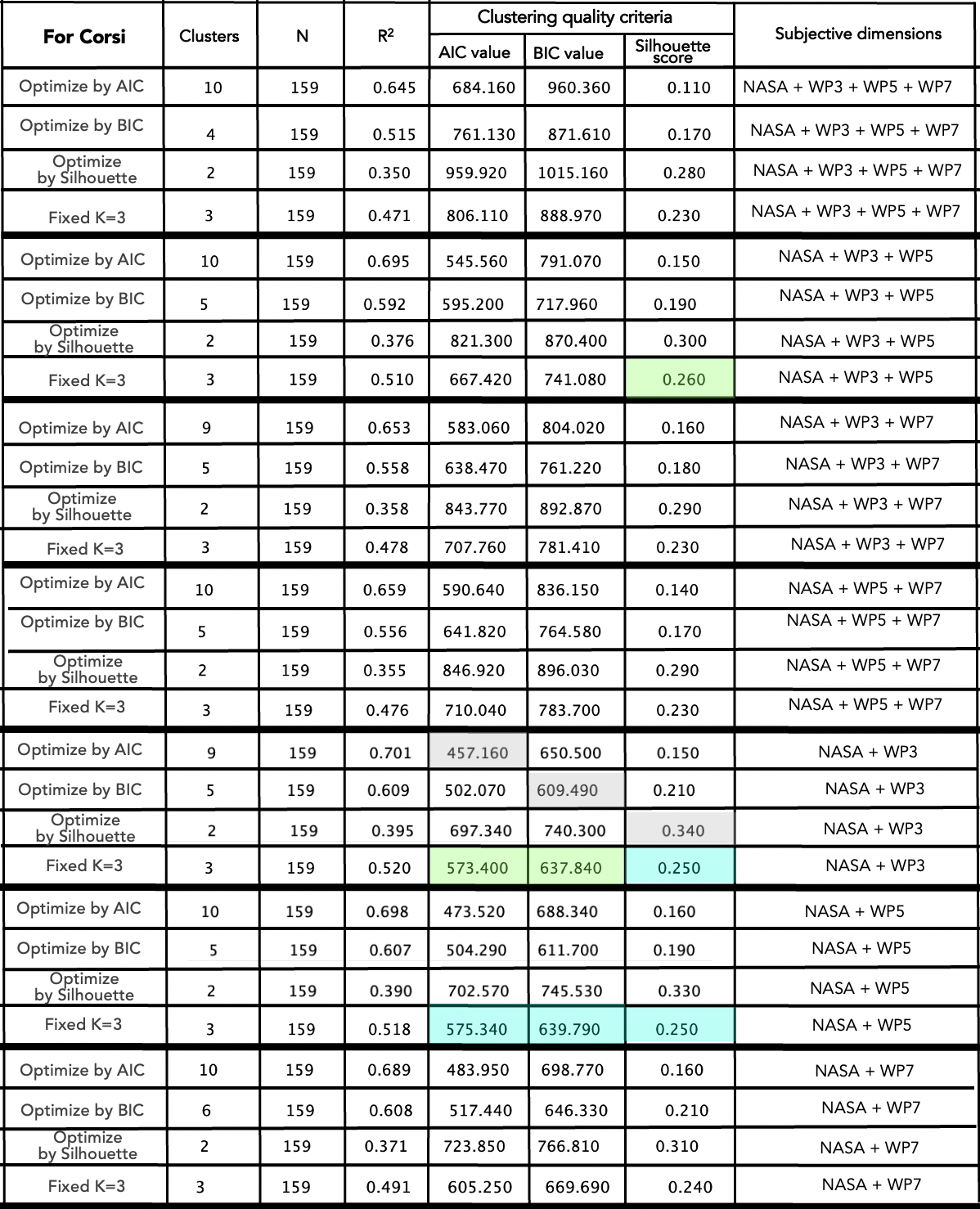


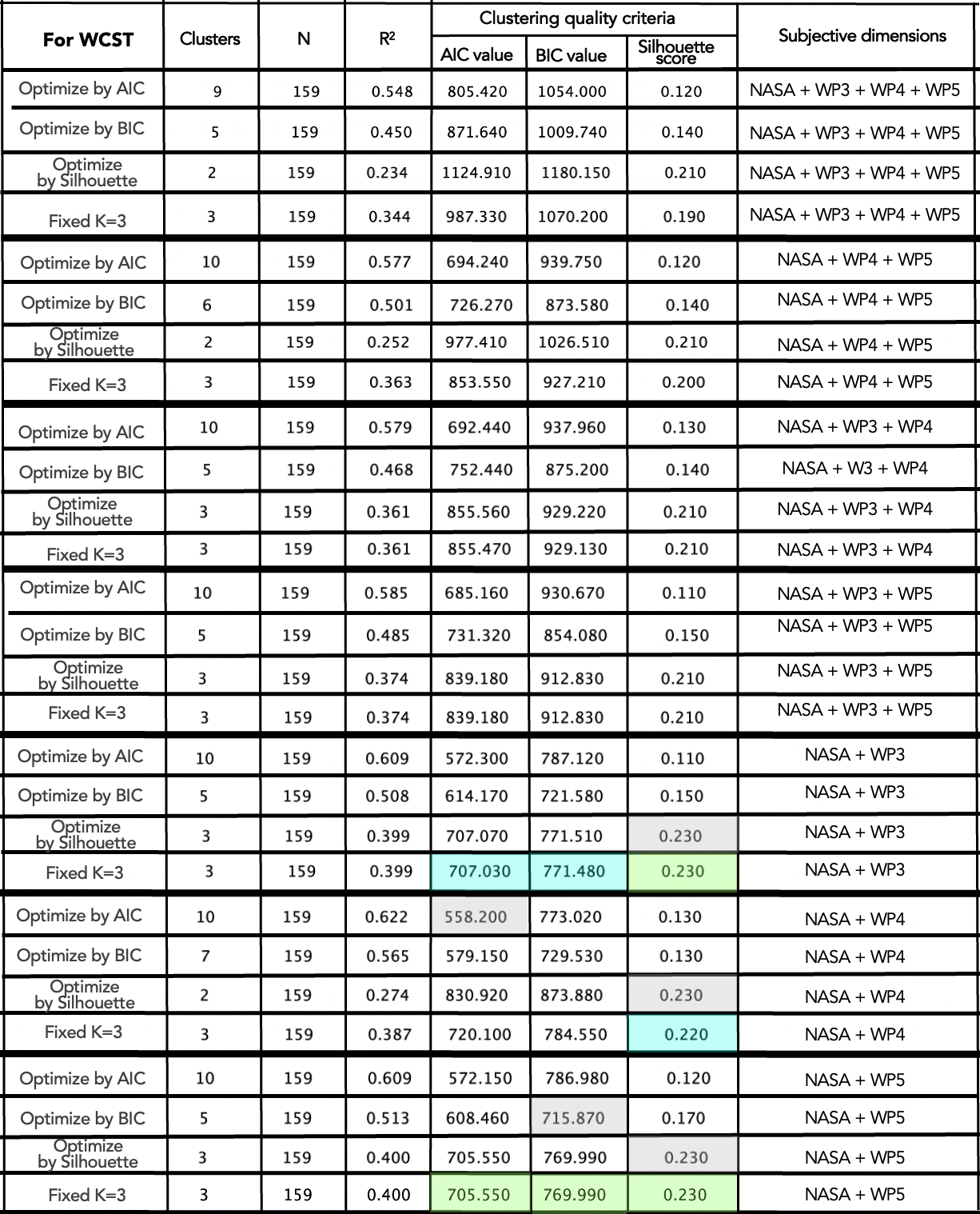


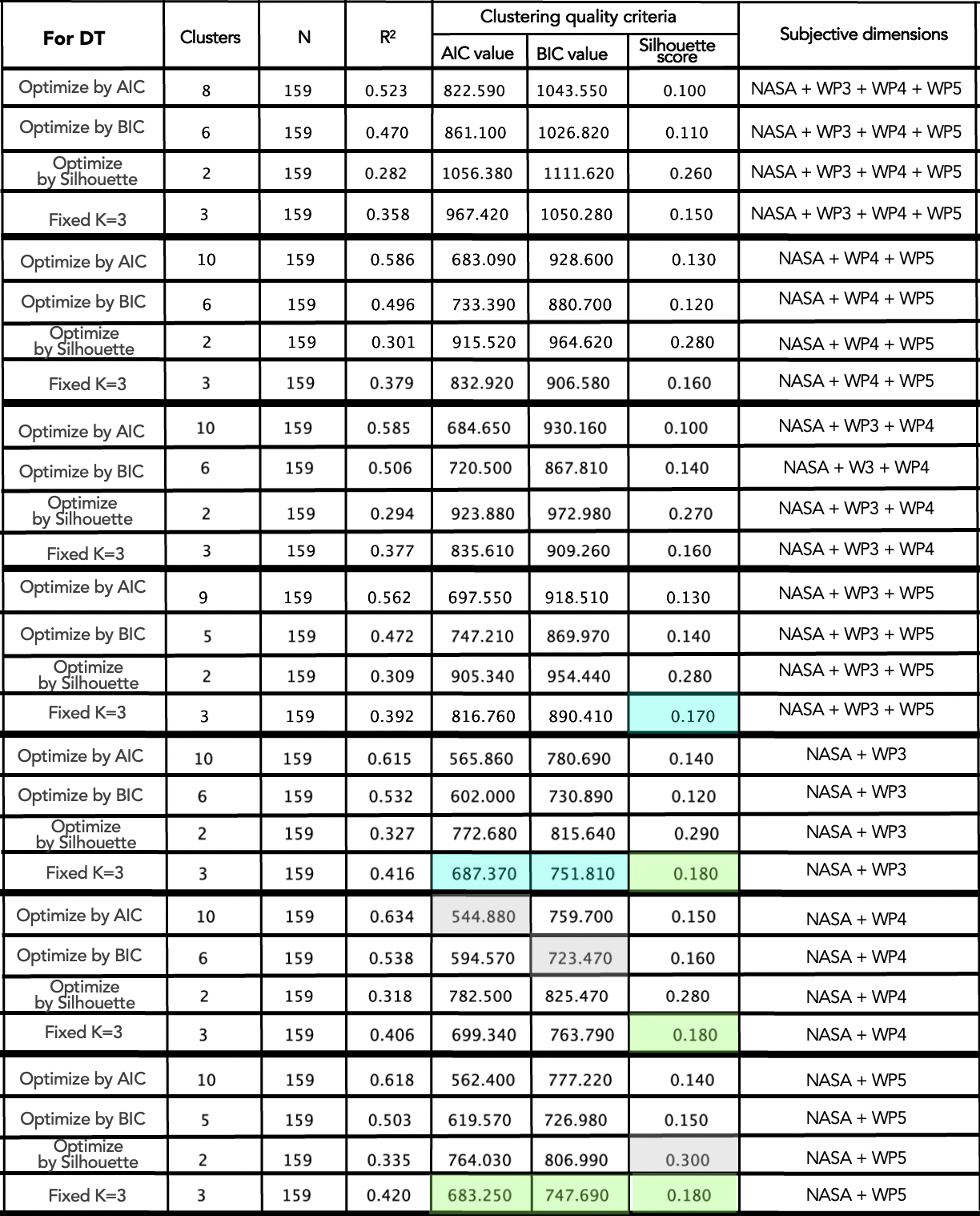


**Supplementary Table 5**: Different K-Means clustering for NBT, GNG, Corsi, WCST and DT. The grayed cells corresponded to the best scores for each clustering quality criteria column (AIC value, BIC value and Silhouette score). For each K=3 of a clustering quality criterion, we had green (the best clustering quality score) and light blue (the second).
